# Analysis of the molecular subtypes and prognostic models of anoikis-related genes in colorectal cancer

**DOI:** 10.3389/fonc.2025.1579843

**Published:** 2025-06-30

**Authors:** Lei Shen, Kang Hou, Jifeng Zhang, Xiaodong Li

**Affiliations:** ^1^ Department of Gastroenterology, Zibo Central Hospital, Zibo, Shandong, China; ^2^ Department of Oncology, Zibo Central Hospital, Zibo, Shandong, China

**Keywords:** colorectal cancer, anoikis, prognostic signature, immune infiltration, gene subtypes

## Abstract

**Background:**

Colorectal cancer (CRC) is a malignant tumor originating from the epithelial cells of colon or rectum. Currently, the main treatment strategy is surgery with chemotherapy and radiotherapy, but the 5-year survival rate is only 63%. Therefore, new therapeutic targets should be discovered and identified to improve survival. This study explored the critical role of anoikis-related genes in CRC development, investigated the regulatory mechanism and identified potential therapeutic drugs using data from the TCGA database, offering a theoretical foundation for CRC diagnosis and treatment.

**Methods:**

Anoikis-related genes differentially expressed in CRC tissues compared to normal tissues were identified using data from the TCGA dataset. Prognostic gene signatures were constructed using both univariate and multivariate Cox regression models. Validation of target gene expression was performed by Western blotting and qRT-PCR. To elucidate the regularity mechanisms underlying the identified gene signature, KEGG, GO, immune infiltration analysis and ssGSEA were conducted. Additionally, various computational algorithms were employed to evaluate the immunotherapeutic responses of different risk groups. The oncoPredict package predicted candidate chemotherapy agents.

**Results:**

Based on screening and identification results, we established three anoikis-related genes: LEP, HAMP, and FAM43B, as the prognostic prediction genes of CRC. We successfully constructed the study model and demonstrated that the risk score of the anoikis-related prognostic prediction signature is an independent prognostic factor in overall survival. Additionally, the results of immune microenvironment infiltration showed that the high-risk score group had a greater infiltration of the M0, M1, and M2 macrophages. In the immunotherapy cohort, the prognosis of patients with a high score, as judged by the study model, was significantly better. The risk score of the anoikis-related prognostic prediction gene is associated with the immunotherapy response in metastatic colorectal cancer.

**Conclusions:**

Our study reports on the identification of anoikis-related gene subtypes and the construction of a prognostic signature in CRC, which, in turn, can provide a basis for further study of the molecular mechanism, clinical diagnosis, and treatment of CRC.

## 1 Background

Colorectal cancer (CRC) is a heterogeneous disease characterized by gene fusion, epigenetic changes, somatic mutation and genetic instability, posing a serious threat to human health (1–4). Approximately 70% of sporadic CRC cases originate from adenomatous polyps, while 25–30% are linked to sessile serrations (5). CRC has a complex and diverse pathogenesis, influenced by various factors such as environmental and dietary factors, lifestyle choices, and familial and hereditary factors (6). At present, surgical resection is the main treatment method for CRC, yet even with the addition of modern adjuvant systemic therapies, only 20% of CRC patients achieve a cure (7). Globally, over 1.85 million CRC cases and 850,000 deaths are reported annually. It is claimed that in 20% of the newly diagnosed CRC cases, the disease has already progressed to the metastatic stage, while it will become metastatic later in the other 25% of the patients (8). Treatment options for metastatic CRC remain limited, with chemotherapy being the conventional method. However, clinically, only a few tumor-targeted chemotherapy drugs are available, such as epidermal growth factor receptor inhibitors and vascular endothelial growth factor inhibitors, and these drugs are effective only in patients with specific mutations (9). Encouragingly, rapid development and in-depth research on molecular biology and genotyping have shifted tumor-targeted therapy from the molecular level to the genetic level, yielding promising results. Thus, there is a pressing need to identify new therapeutic targets for CRC to fit in the targeted therapy development.

Loss of adhesion or improper adhesion between cells and the extracellular matrix (ECM) is known as “nesting.” Once normal epithelial cells lose contact with the ECM, they rapidly undergo anoikis, a specific form of apoptosis first described in 1994 by Frisch et al. Endothelial and normal epithelial cells are typically adhesion-dependent, relying on signal transmission between cells and the ECM for survival, which is a phenomenon known as anchoring dependence (10). For example, when normal epithelial cells and solid tumor cells without metastatic properties lose their intercellular connection and basement membrane support, they detach from their original site and enter the bloodstream, leading to apoptosis. This type of cell apoptosis that breaks away from the original living environment is called anoikis, and it plays a crucial role in maintaining stability and structural integrity. It is essential for the entire process of organism genesis, development, renewal, and degradation to prevent the exfoliated cells from planting and growing in unsuitable places (11). However, malignant tumor cells evade anoikis, allowing them to migrate and proliferate in new sites after shedding from the primary tumor. Anoikis is closely associated with tumor progression and prognosis, making it a potential target for anti-cancer therapies. Despite this, the role of anoikis-related genes in the development of common malignant tumors of the digestive tract in CRC remains unclear.

This study applies the CRC data from the TCGA database to investigate the anoikis-related gene expression to stratify patients and construct anoikis-related subtypes. Moreover, it explores the regulatory mechanism and potential targeted therapies, providing new insights into the molecular mechanism, clinical diagnosis, and treatment of CRC.

## 2 Materials and methods

### 2.1 Acquisition and processing of training dataset

In this study, data integration and extraction of CRC samples were performed using the TCGA Target GTEX dataset available in the UCSC Xena database (12). Survival information for CRC samples was retrieved from the integrated data on cell literature (13). Ultimately, 698 RNA-seq expression samples were prepared for this research, including 51 normal intestinal tissues and 647 CRC samples, along with clinical information of 630 patients. The clinical information statistics of patients (training set) are shown in **Supplementary Figure 4**.

### 2.2 Acquisition and processing of validation queue data

GSE39582 (https://pubmed.ncbi.nlm.nih.gov/23700391/), containing microarrays of CRC tissue samples to verify the prediction model, was used in this study. These microarrays were sourced from the GPL570 (Affymetrix Human Genome U133A array) platform and initially analyzed using GEO2R. All data were processed in R software (version R 4.1.2) the limma package (limma 3.26.8) and Biobase 2.30.0 (14). A log transformation was applied to the original data, followed by a *t*-test. GEO2R was used to compare the submitter-supplied data, with fold-change significance set at *P* < 0.05 and a threshold of >2.0 or < –2.0. If duplicate genes were encountered, the median expression value was taken as the final expression amount. During data processing, the no-load probe was first deleted. Probs corresponding to multiple genes were also deleted. The median expression value was taken as the expression value of the gene for multiple probes mapping to the same gene.

### 2.3 Acquisition and processing of anoikis-related genes

The anoikis-related gene list was downloaded from the MSigDB database (https://www.gsea-msigdb.org/gsea/msigdb/) on the file “msigdb.v7.4.symbols.gmt” by searching for the term “anoikis” under Gene Ontology Biological Process (GOBP)-related pathway genes. After removing the duplication genes, 34 genes were included in the analysis.

The HALLMARK pathway gene set (msigdb.v7.4.symbols.gmt) was also downloaded from the MSigDB database. Genes with a degree of >2 (connectivity between a single gene and other genes) were selected for subsequent cluster analysis. A total of 21 genes were identified: AKT1, SRC, MTOR, NOTCH1, MCL1, PIK3CA, PTK2, ITGB1, TSC2, STK11, CAV1, SNAI2, ITGA5, CHEK2, E2F1, IKBKG, NTRK2, BCL2, CEACAM6, PTRH2, and TLE1.

MuTect1 software with default parameter values and IndelGenotyper (https://software.broadinstitute.org/gatk/) was applied for variant calling (15). Each tumor sample was paired with a corresponding paracancerous tissue to filter out any variations common to both the tumor and the normal sample.

Unsupervised clustering analysis was performed to cluster patients into different molecular subtypes by R package “ConsensusClusterPlus” according to the anoikis-related genes expression. The following criteria were applied during clustering: (1) increased intra-group correlation with decreased inter-group correlation following clustering; (2) no cluster with a small sample size, and (3) a gradual and smooth increase in the cumulative distribution function (CDF) curve. Gene set variation analysis (GSVA) used the hallmark gene set (c2.cp.kegg.v7.2) derived from the MSigDB database to explore differences in biological processes related to anoikis genes among clusters.

### 2.4 Model building

The up-regulated genes were treated as clusters’ markers. After identifying the clustering subtypes, limma v3.26.8 was applied to perform differential gene expression analysis among the subtypes, with a threshold FDR < 0.05 and |log2FC| > 2 to identify differentially expressed genes. In total, 477 identified genes were selected for subsequent analysis; 2 genes were in cluster 1 and the rest 475 genes were in cluster 2. These genes were further analyzed using univariate Cox regression analysis with a significance threshold of *P* < 0.01. As a result, 47 genes were identified as significant prognostic factors; their up-regulation was associated with poorer outcomes.

Next, the least absolute shrinkage and selection operator (Lasso) regression algorithm was applied to identify the genes significantly associated with the prognostic factors through the “glmnet” package in R. This analysis yielded three signature genes, which were used to construct a prognostic model. The model’s formula was as follows:


$$\text{Risk Score}=\sum_{i=1}^{n}{\text{Expression}}_{i}\times {\text{Coefficient}}_{i}\;$$

where ${\text{Expression}}_{i\;}\;$ presents the amount of expression of each gene, and ${\text{Coefficient}}_{i}$ means the weight coefficient of each gene.

Survival analysis was conducted using the “surveyors” package in R to assess the prognostic difference between the high and low-score groups, which was categorized by the median score value. Principal component analysis (PCA) was performed to visualize the transcriptome profiling differences between the groups.

### 2.5 Functional enrichment analysis

The Cytoscape (v3.9.1) software was employed to draw the Protein–Protein Interaction (PPI) network of anoikis-related genes. R package “clusterProfiler” (16) was employed to perform gene oncology (GO) enrichment analysis with a significance threshold of *P*-value < 0.05. CRC patients were classified into various clusters to explore the functional roles of CRC-related genes by “ConsensusClusterPlus” package in R with 80% resampling rate Pearson correlation and 50 iterations. Additionally, PCA was applied to analyze gene-expression profiles in both the high- and low-score CRC groups.

### 2.6 Sample collection and processing

Tumor and paired adjacent tissues from the patients (*n* = 26) were obtained from ZIBO CENTRAL HOSPITAL (Zibo, Shandong, China). None of the donors had taken any drugs or hormones prior to surgery. The ethics review committee of the ZIBO CENTRAL HOSPITAL was informed of the process and due approval was obtained.

All tissue samples were collected from the discarded tissues in surgery. After collection, the samples were quickly frozen in liquid nitrogen and stored at −80°C freezers. RNA was extracted from the snap-frozen samples by placing them in precooled TRIzol reagent (Thermo Fisher Scientific, Waltham, MA, USA), followed by chloroform extraction and isopropanol precipitation. Then, the extracted RNA was quantified with a spectrophotometer. The proteins were extracted using the ProteoExtract Native Protein Extraction Kit (Thermo Fisher Scientific, Waltham, Massachusetts, USA), according to the manufacturer’s instructions, and the extracted protein concentrations were measured using the Quick Start Bradford protein assay.

### 2.7 Verification of the expression of target genes

Protein preparation, Western blotting, RNA isolation, and qRT-PCR were performed as previously described (17). The following primers were used for RT-PCR: LEP, sense 5′-GCCCAGCAACATTAT CCAGT-3′ and anti-sense 5′-AGCCCTTTCTCAAAGGCTTC-3′; HAMP, sense 5′-CACAACAGAC GGGACAACTT-3′ and anti-sense 5′-CGCAGCAGAAAATGC AGATG-3′; and FAM43B, sense 5′-AGGGTAAGGGGAGGGGATA A-3′ and anti-sense 5′-CCTAAAAATACC CAA TACCAAACA-3′.

The rabbit monoclonal anti-Leptin (LEP) antibody (ab16227), rabbit monoclonal anti-Hepcidin-25 antibody (HAMP) (ab238974), and rabbit monoclonal anti-FAM43B antibody (ab121299) were purchased from Abcam (Cambridge, UK). The mouse anti-rabbit IgG-horseradish peroxidase (HRP)-conjugated secondary antibody (1:5000 in TBST) was purchased from Soleibao (Beijing, China). The specific detailed steps are provided in the **Supplementary Material**.

### 2.8 Single sample gene set enrichment analysis

Single sample gene set enrichment analysis (ssGSEA) was used to calculate the normalized enrichment score (NES) score in each channel of each sample in the HALLMARK gene set, implemented in the GSVA R package (18–20). The enrichment analysis of differentially up-regulated genes among the subtypes was completed by the clusterProfiler R package, with significance thresholds set to pvalueCuttoff=0.05, pAdjustMethod=“BH”.

### 2.9 Immune infiltration analysis

The ESTIMATE algorithm was applied to calculate the stromal score, immune score, and ESTIMATE score (21). Immune cell infiltration, comprising 22 infiltrating immune cell types, was assessed using the CIBERSORT algorithm (parameter settings: perm=200, arrays=FALSE; other parameters were set by default), which was integrated into the IOBR R package (version 3.6.5) (22).

### 2.10 Prediction of the potential chemotherapeutic agents and immunotherapy cohort

We downloaded the gene-expression profiles and the corresponding drug response data from the Genomics of Drug Sensitivity in Cancer 2 (GDSC2) database using oncoPredict package in R (23). Sensitivity scores were then generated using oncoPredict package to predict the half-maximal inhibitory concentration (IC_50_) of all drugs in CRC patients. Data were downloaded from The IMvigor210 using the IOBR package (24, 25), including tumor mutation burden, therapeutic responses, gene-expression profiles, survival data, and neoantigen information.

### 2.11 Statistical analysis and other details

R software version 4.1.2 was used for the statistical analysis. Data integration and mapping were performed using the tidyverse package (26). The Kaplan–Meier curves were employed to assess the differences in the overall survival (OS) between the high- and low-score CRC patient groups, with the log-rank tests for significance test. To compare continuous and ordered categorical variables, we applied the Wilcoxon test and the Kruskal–Wallis test. The assessed variables include score distribution differences between clinical features, differences in the HALLMARK NES scores between the clusters, and immune cell infiltration proportions between different sub-rents. The Fisher exact test was used to compare unordered categorical variables, such as the distribution of immunotherapy patients among different scoring groups. Heat maps were generated using the ComplexHeatmap R package, while gene correlation maps were visualized with the corrplot R package (27, 28). The single or multifactor forest map was drawn by using the forestmodel R package (29). *P* <.05 were considered statistically significant for all analyses. Statistical test marks: *P <.05, **P <.01, ***P <.001, ***P <.0001. NS: not significant.

## 3 Results

### 3.1 Landscape of anoikis-related gene changes

**Figure 1:**
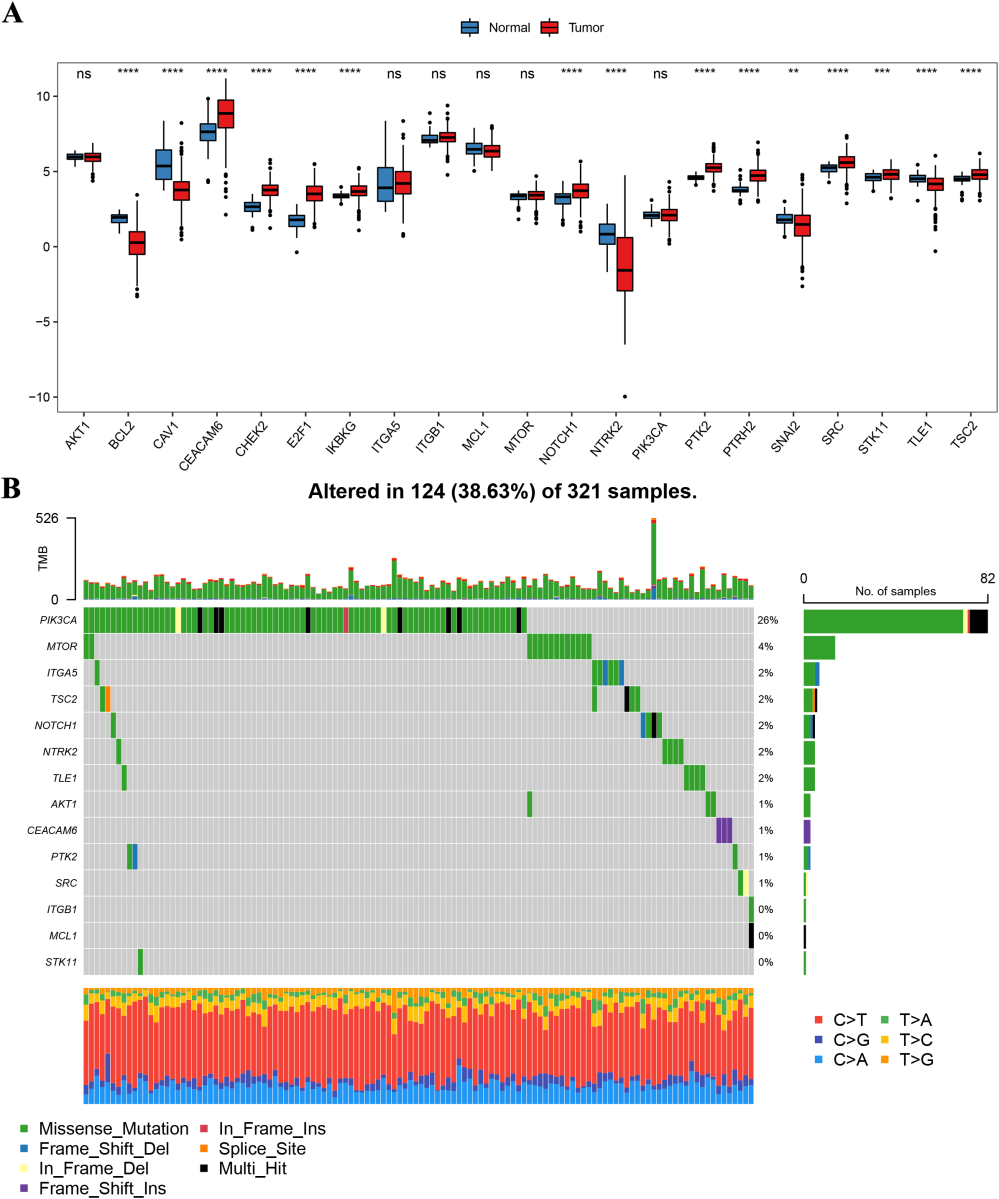
Landscape of gene expression differences and mutations in anoikis-related genes. **(A)** Box plot comparing the expression levels of anoikis-related genes between normal adjacent tissues (blue) and cancerous tissues (red) in CRC patients. **P <.01, ***P <.001, ****P <.0001. NS, not significant; **(B)** Summary of somatic mutations in anoikis-related genes in CRC patients from the TCGA-COADREAD cohort (321 patients).

In the training set samples, we compared the 21 anoikis-related gene expression between CRC patients and normal adjacent cancers and found that the expression levels of CEACAM6, CHEK2, E2F1, IKBKG, NOTCH1, PTK2, PTRH2, SRC, STK11, and TSC2 were higher in cancerous tissues than they were in normal adjacent tissues (**Figure 1A**). Besides, we compared the anoikis-related gene expression across CRC patients with varying clinical characteristics and found that the MCL1 expression was higher in the older age group. CAV1, CHEK2, IKBKG, ITGA5, ITGB1, PTK2, SNAI2 were higher, while PTRH2 was lower in the invasion-existing group than they were in the invasion-absent group. TLE1 was higher in the group with the primary lymph node compared to the group without it. BCL2 and CHEK2 were lower and IKBKG, ITGA5, NTRk2, PTK2, SRC, and TSC2 were higher in grade III/IV comparing to grade I/II. IKBKG was higher in the group with the Venous-Under the condition of invasion than that without it (**Supplementary Figure 1**).

A summary analysis of somatic mutation incidence within these 21 anoikis-related genes in the TCGA-COADREAD queue showed that 14 of these genes exhibited relatively high mutation frequency. Among the 321 samples in the TCGA-COADREAD queue, 124 (38.63%) showed mutations in the anoikis-related genes, with PIK3CA showing high mutation frequency, while other genes displayed lower mutation rates (**Figure 1B**). The chromosomal location distribution of these anoikis-related genes is presented in **Supplementary Figure 2** and the corresponding volcano map is shown in **Supplementary Figure 3**.

### 3.2 Regulation patterns of different anoikis-related genes in tumors

**Figure 2:**
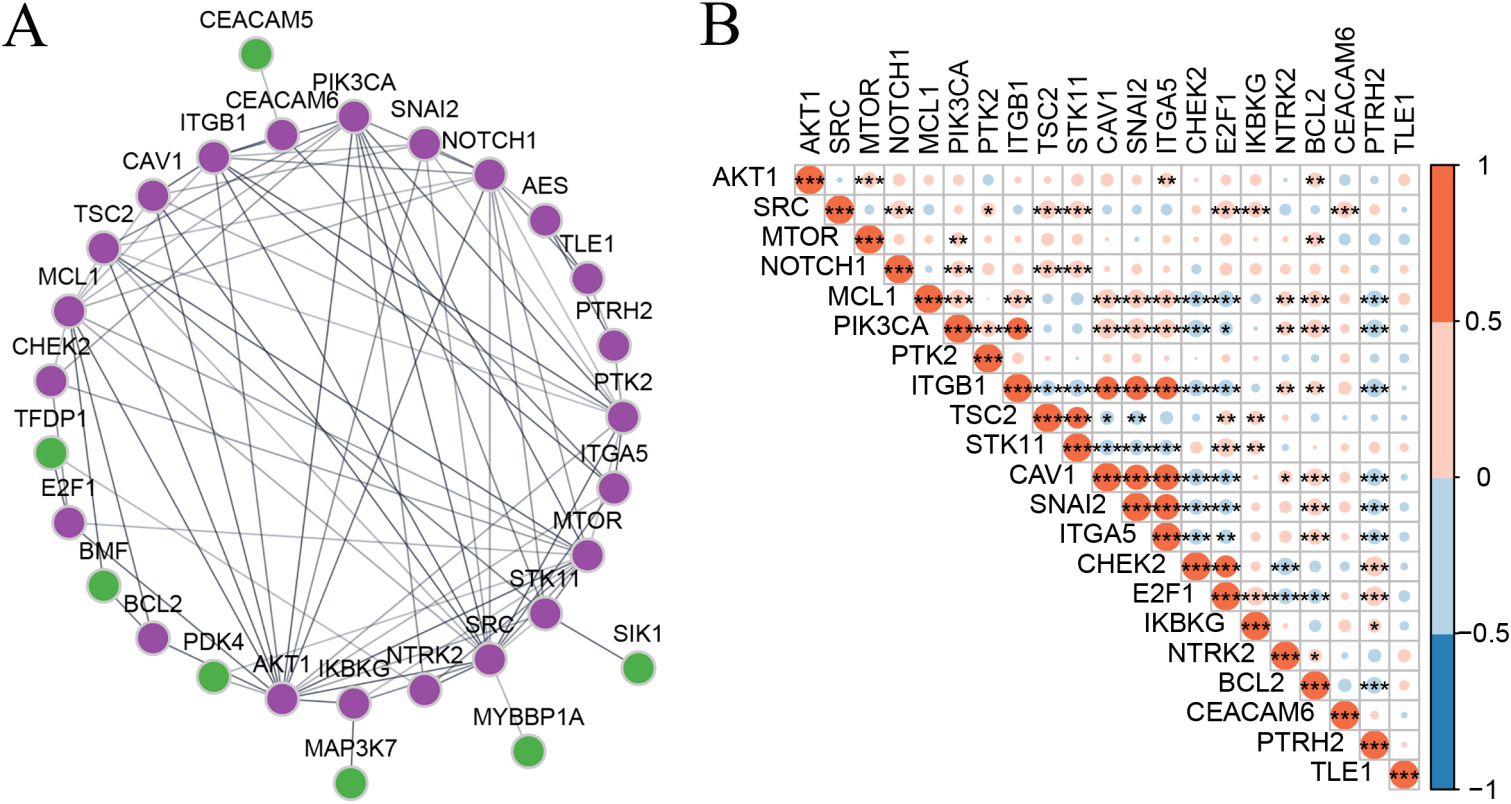
Regulation patterns of different anoikis-related genes in tumors. **(A)** PPI network of anoikis-related genes, with genes marked in purple included in the clustering analysis. **(B)** Spearman correlation matrix of anoikis-related gene expression in CRC. Statistical test marks: *P <.05, **P <.01, ***P <.001, ***P <.0001. NS, not significant.

To explore the interactions among these anoikis-related genes, we constructed a PPI network from the String database, with isolated nodes removed. The analysis revealed that AK1 and PIK3CA were closely related to other genes (**Figure 2A**). SRC and TSC2, MCL1, and CAV1 showed significant correlation based on their expression (**Figure 2B**). GO enrichment analysis results showed that these genes are significantly enriched in pathways related to anoikis, focal induction, and protein heterodimerization activity, which further confirmed that the screened genes were anoikis-related genes (**Supplementary Figure 4**).

**Figure 3:**
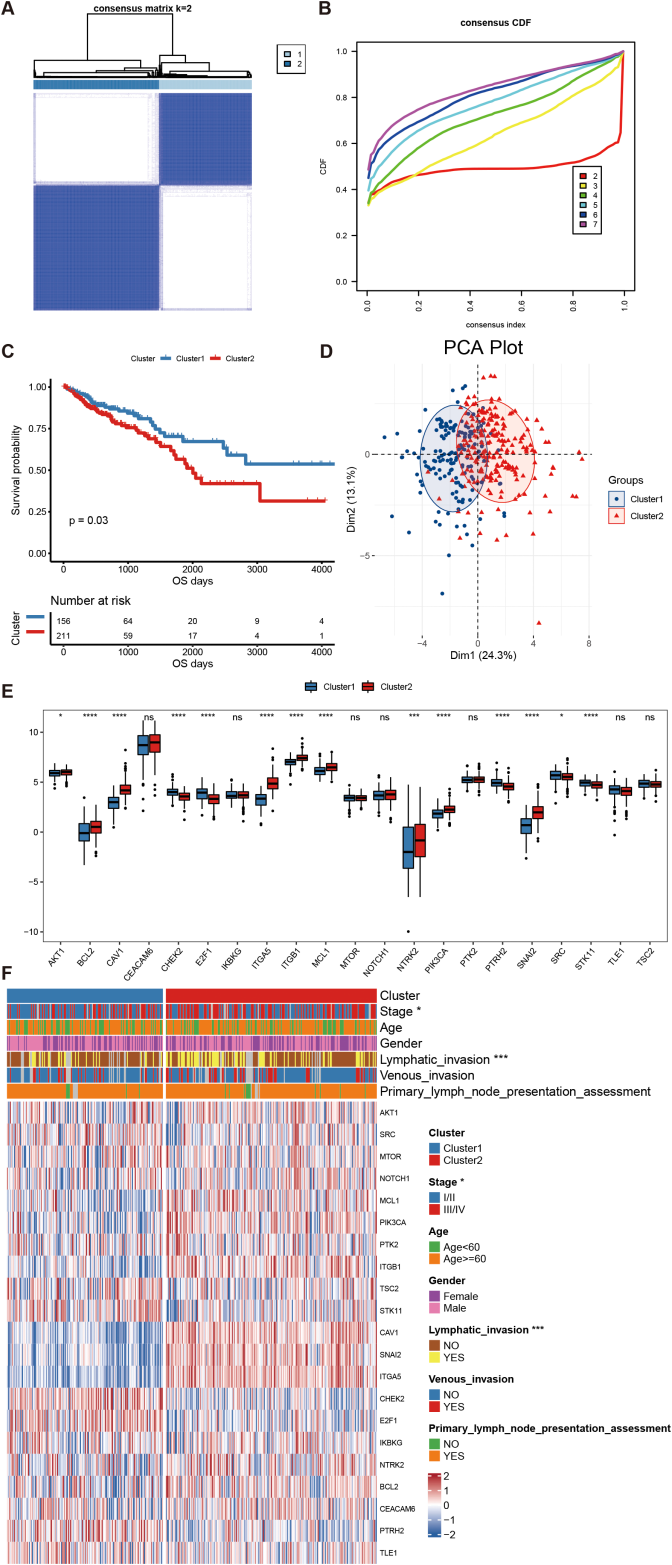
Cluster results of anoikis genes in CRC. **(A)** Thermal graph of sample classification based on cluster analysis. **(B)** Consensus clustering CDF curves for *k* values from 1 to 9. **(C)** Kaplan-Meier curves showing the outcome difference among patients with different subtypes. **(D)** Principal component analysis of the expression of anoikis-related genes in the Target GTEX dataset. **(E)** Box plot comparing the expression levels of anoikis-related genes between different cuproptosis subtypes. **(F)** Heatmap illustrating the differential expression of anoikis-related genes in different subtypes and clinical characteristics of patients. *P<.05, ****P<.001, ***P<.0001. NS, not significant.

We performed cluster analysis on CRC samples in the training set queue on the expression of these anoikis-related genes. The optimal number of clusters was determined to be *k* = 2, as indicated by the CDF decline curve and the cluster sample distribution heat map (**Figures 3A, B**). The cluster1 subtype demonstrated a significantly better OS outcome (HR = 0.68, log-rank *P* <.05, **Figure 3C**). The expression of anoikis-related genes in cluster 1 and cluster 2 subgroups were compared using the principal component analysis (PCA). The results suggested that there were significant differences between cluster 2 and cluster 1 (**Figure 3D**). Next, we characterized the anoikis-related gene expression between the subtypes and verified the statistical significance using the Kruskal–Wallis rank-sum test, revealing significant differences in gene expression (**Figure 3E**, marked with an asterisk). Positively correlated genes displayed similar expression patterns, whereas negatively correlated genes exhibited inverse expression trends. **Figure 3F** shows the expression heat map and the clinical characteristics heat map of each cuproptosis-related subgroup of patients. These results indicate that the two clusters were closely related to CRC.

### 3.3 Immunoregulatory mechanism of different molecular subtypes of anoikis-related genes

**Figure 4:**
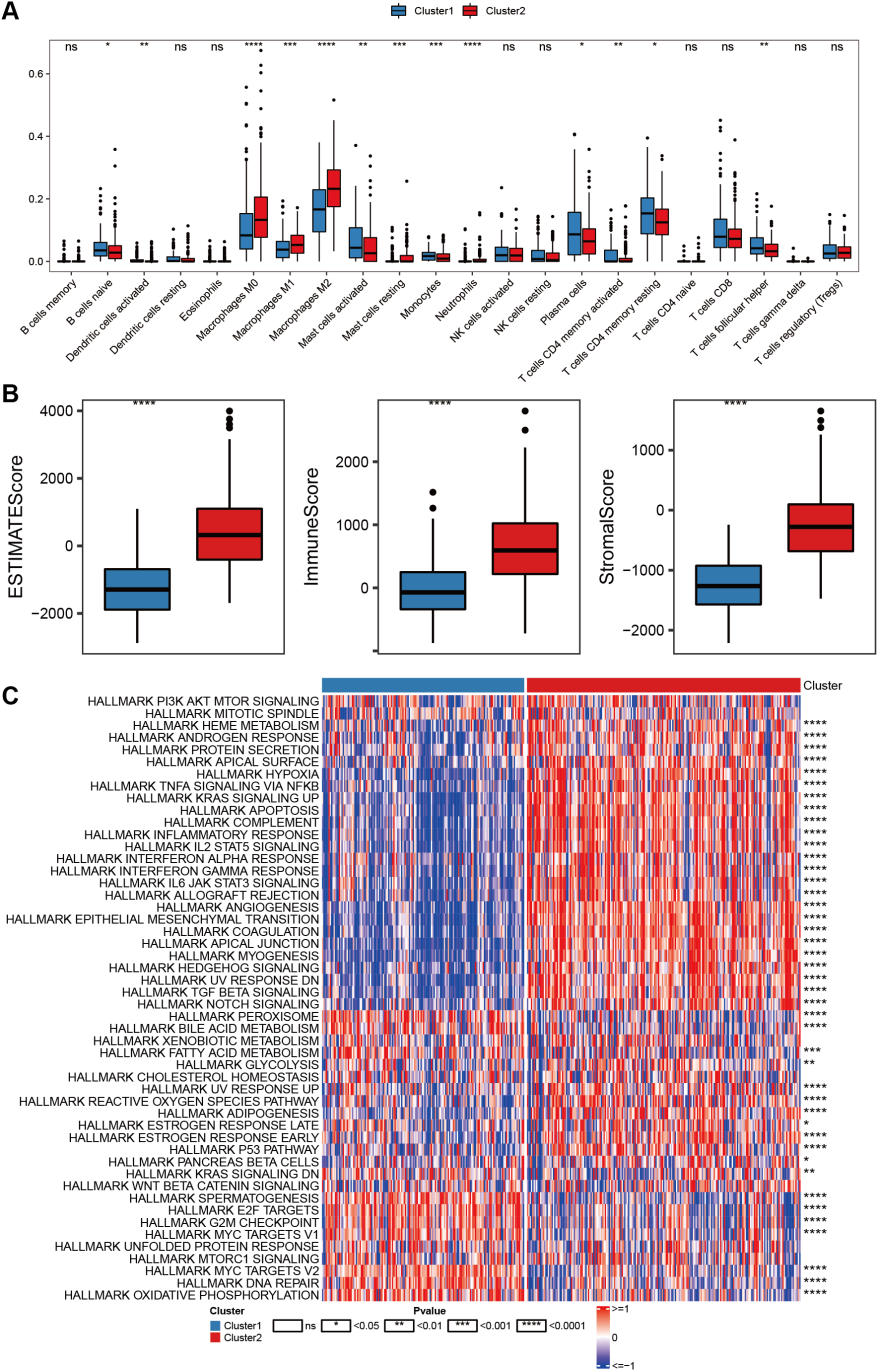
Immunoregulatory mechanism of different molecular subtypes of anoikis-related genes. **(A)** Box plot comparing the scores of different cellular immune infiltration proportions between Cluster 1 (blue) and Cluster 2 (red) using the CIBERSORT algorithm. **(B)** Box plots illustrating the comparison of ESTIMATE scores, immune scores and stromal scores between Cluster 1 (blue) and Cluster 2 (red) using the ESTIMATE algorithm. **(C)** Heatmap of hallmark pathway enrichment scores (ssGSEA scores) across CRC subtypes calculated by the GSVA R package. Statistical test marks: *P <.05, **P <.01, ***P <.001, ***P <.0001, ****P<.0001. NS, not significant.

After determining the relevant subtypes of CRC by using anoikis-related genes, we employed the CIBERSORT algorithm to compare immune cell infiltration between Clusters 1 and 2 (**Figure 4A**). The proportion of plasma cells and other immune cells was significantly lower in Cluster 2 than in it was Cluster 1 (*P* = 0.0003). At the same time, we further calculated differences in the immune scores, ESTIMATE scores, and matrix scores of the clusters using the ESTIMATE algorithm. Cluster 2 exhibited significantly lower tumor purity scores compared to that in Cluster 1 (**Figure 4B**).

Moreover, we utilized the HALLMARK pathway data from the MSigDB database to calculate the enrichment scores (ssGSEA scores) of different CRC subtypes by using the GSVApackage. The significance between the groups was determined by the rank sum test. Cluster 1 was observed to be positively correlated with most cancer super-pathways (rank-sum test, *P* = 0.00005). Specifically, oncogenic pathways such as DNA repair (rank-sum test, *P* = 0.00006), MYC targets V1 (rank-sum test, *P* = 0.00004), and MYC targets V2 (rank-sum test, *P* = 0.00002) showed a strong correlation with Cluster 1. Conversely, several pathways such as HEME metabolism (rank-sum test, *P* = 0.00007), ANDROGEN response (rank-sum test, *P* = 0.00003), and HYPOXIA pathways (rank-sum test, *P* = 0.00002) were negatively correlated with Cluster 1.

In contrast, Cluster 2 was positively correlated with most immune response super-pathways (rank-sum test, *P* = 0.00006). Among these pathways, immune pathways such as IL6, JAK, and STAT3 signaling (rank-sum test, *P* = 0.00003); IL2, STAT5 signaling (rank-sum test, *P* = 0.00008); and interferon-gamma response (rank-sum test, *P* = 0.00006) showed a strong correlation with Cluster 2. Pathways negatively correlated with Cluster 2 included bile acid metabolism, spermatogenesis, and MYC targets V1, as shown in **Figure 4C**.

### 3.4 Analysis of the regulation model of different molecular subtypes of anoikis-related genes

**Figure 5:**
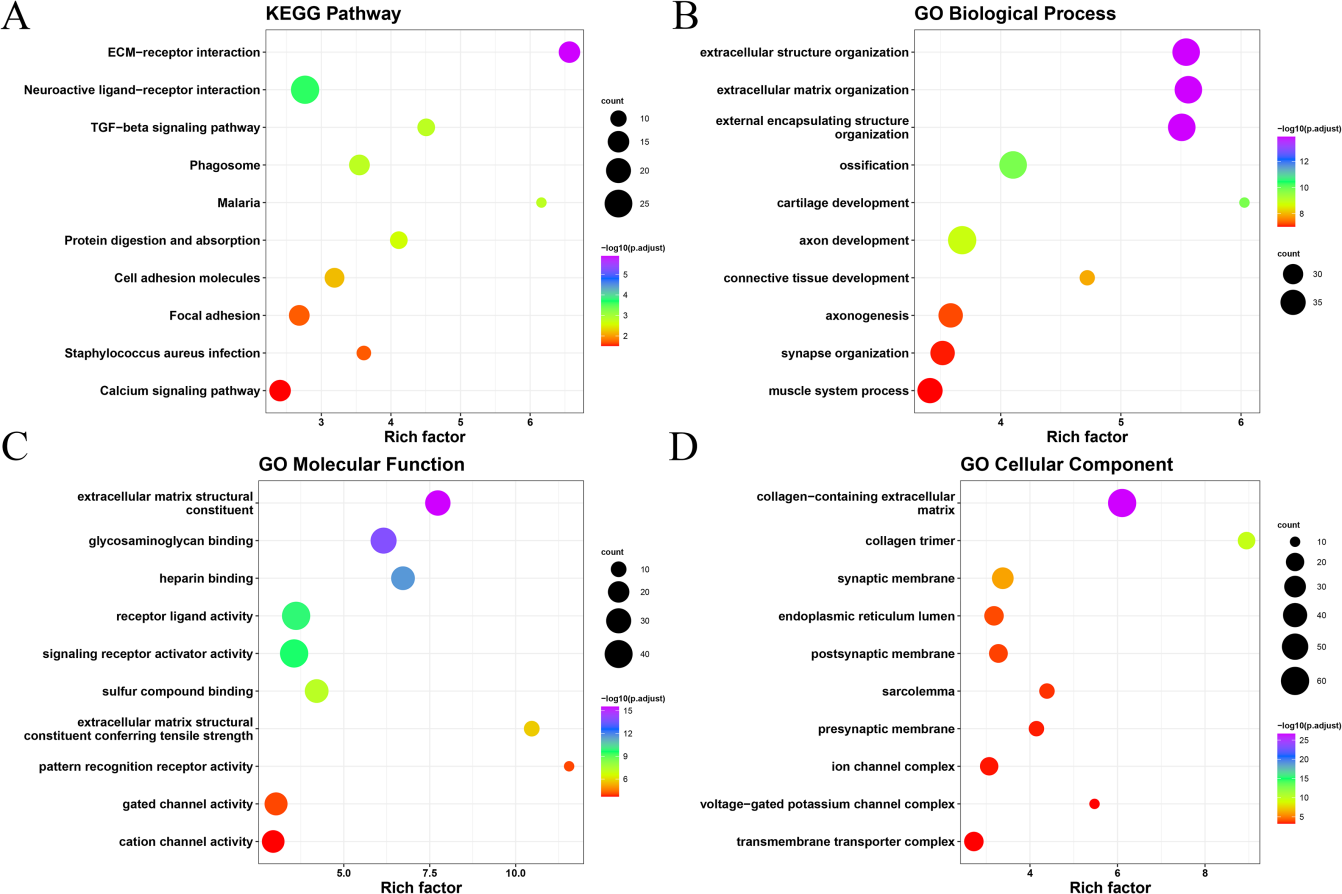
Results of GO and KEGG enrichment analysis of anoikis-related genes of CRC: **(A)** KEGG enrichment analysis; **(B)** GO biological process; **(C)** molecular function; and **(D)** cellular component.

After exploring differences in the immune mechanisms among the subgroups, we further analyzed the differentially expressed genes among subtypes. A total of 477 significantly differential genes expressed among subtypes were selected for KEGG and GO enrichment analyses (**Figure 5**). KEGG pathway enrichment revealed that these genes were significantly enriched in ECM–receiver interactions, neuroactive light–receiver interactions, and other pathways. While GO enrichment showed that these genes were associated with external encapsulating structure organization, extracellular structure organization, extracellular matrix organization, extracellular matrix structural constituent, collagen-containing extracellular matrix, glycosaminoglycan binding, heparin-binding, collagen trimer, and so on.

### 3.5 Construction of anoikis-related gene scoring system

**Figure 6:**
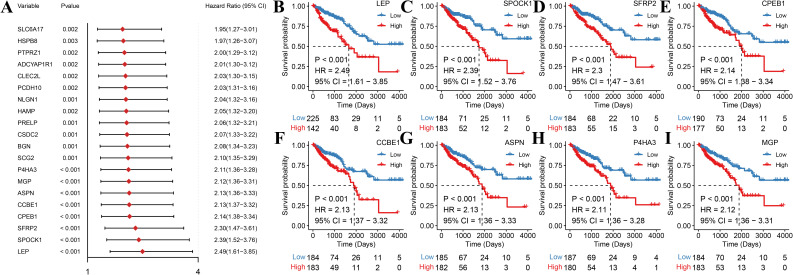
Identification and survival analysis of significant prognostic factors. **(A)** Forest plot of univariate Cox regression for the top 20 differentially expressed genes, showing hazard ratios (HR) and 95% confidence intervals (CI). **(B–I)** Kaplan–Meier survival curves for the top eight genes (LEP, SPOCK1, SFRP2, CPEB1, CCBE1, ASPN, P4HA3, and MGP) with the lowest *P*-values. Patients were divided into high-expression (red) and low-expression (blue) groups according to the median expression threshold.

Among the selected 477 differentially expressed genes, we used the up-regulated genes across subtypes to conduct univariate Cox regression analysis and screened with threshold *P* < 0.01. This yielded 47 genes that might be significant prognostic factors, and a forest plot was generated to show the top 20 genes with the lowest *P*-value (**Figure 6A**). The high- and low-expression groups were determined with the median expression value as the threshold. The top eight genes with the most significant *P*-values were selected to generate the Kaplan–Meier plots (**Figures 6B–I**).

**Figure 7:**
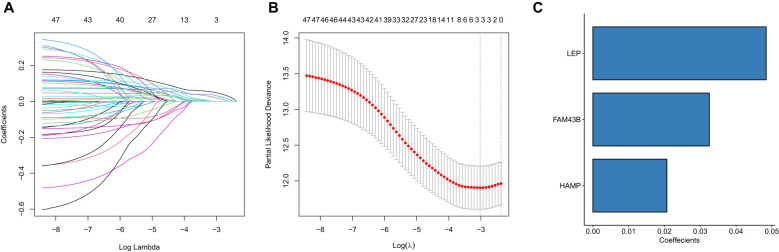
Lasso regression analysis for the identification of prognostic signature genes. **(A)** Lasso coefficient profiles of 47 selected genes. Each track represents a gene, with the *x*-axis indicating the log value of lambda, the *y*-axis indicating the coefficient of the independent variable. **(B)** Tenfold cross-validation for tuning the parameter selection in the Lasso model. The partial likelihood of deviance is plotted against log lambda. The red dots show the mean deviance and the dotted vertical lines represent the confidence interval for each lambda. **(C)** Bar plot showing the regression coefficient of the three signature genes selected by the Lasso regression model.

**Figure 8:**
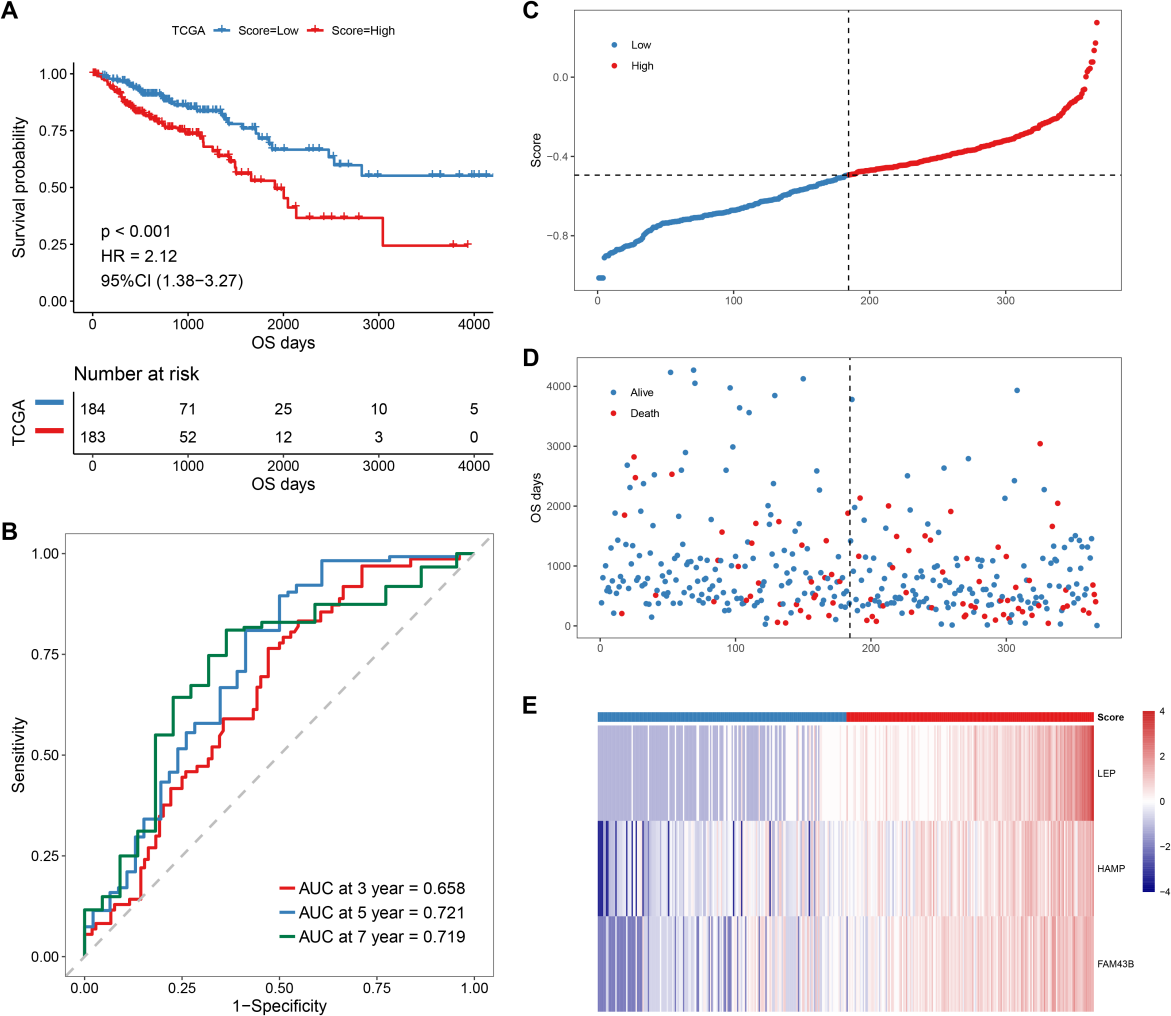
Prognostic analysis of the anokis-related gene score in TCGA CRC patients. **(A)** Kaplan–Meier survival curve for OC in high- and low-score groups. **(B)** Time-dependent receiver operating characteristic (ROC) curve evaluating the predictive accuracy of the prognostic model for 3-year, 5-year, and 7-year survival rates. **(C)** Score distribution of each sample in the dataset (high-score patients in red and low-score patients in blue). **(D)** Scatter plot showing the distribution of survival times in each sample in the dataset. Red dots indicate a decreased number of patients, while blue dots represent survivors. **(E)** Heat map showing the gene expression levels of the three Lasso-selected signature genes in the high- and low-risk groups, with corresponding risk scores.

**Figure 9:**
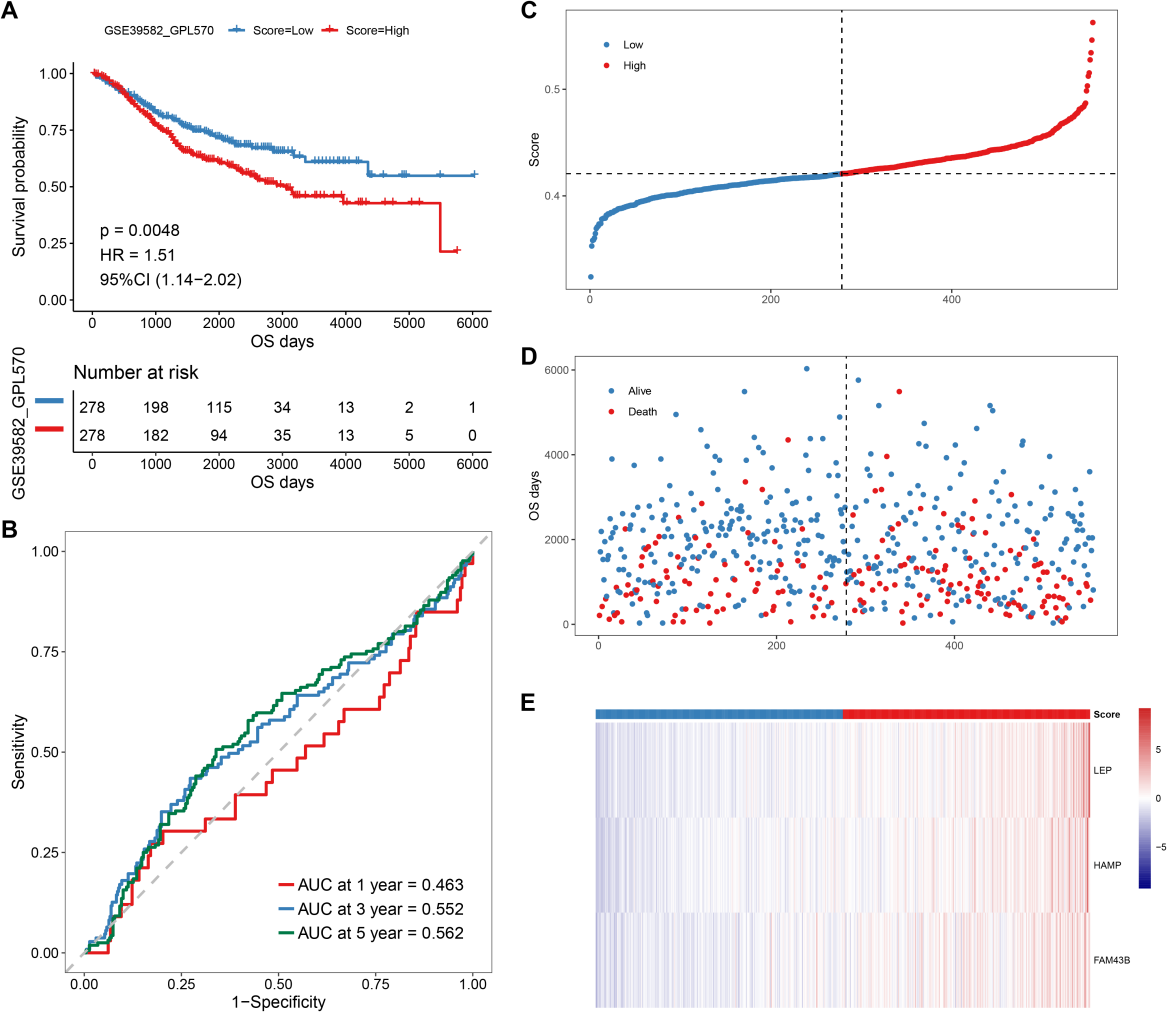
Prognostic analysis of the anokis-related gene score in GSE39582 CRC patients. **(A)** Kaplan–Meier survival curve for OC in high- and low-score groups. **(B)** Time-dependent receiver operating characteristic (ROC) curve evaluating the predictive accuracy of the prognostic model for 3-year, 5-year, and 7-year survival rates. **(C)** Score distribution of each sample in the dataset (high-score patients in red and low-score patients in blue). **(D)** Scatter plot showing the distribution of survival times in each sample in the dataset. Red dots indicate decreased patients, while blue dots represent survivors. **(E)** Heat map showing the gene expression levels of the three Lasso-selected signature genes in the high- and low- risk groups, with corresponding risk scores.

Thereafter, we performed Lasso regression on the 47 significant prognostic factors and identified three signature genes (LEP, HAMP, and FAM43B) to establish a prognosis model (**Figure 7**). The score of each patient was calculated based on the gene expression in the tissue by using the above model formula. The patients were then placed in high- or low-score groups based on the median score. The survival analysis revealed that patients with higher scores had significantly lower OS compared to those with low scores (**Figure 8**). To validate the model, we calculated the scores of each sample using the GSE39582 dataset and divided the TCGA-COADREAD sample into high- and low-score groups based on the median score. Similar to the results obtained for the TCGA queue, patients with high scores in the GSE39582 dataset have worse outcomes compared to those with low scores (**Figure 9**).

**Figure 10:**
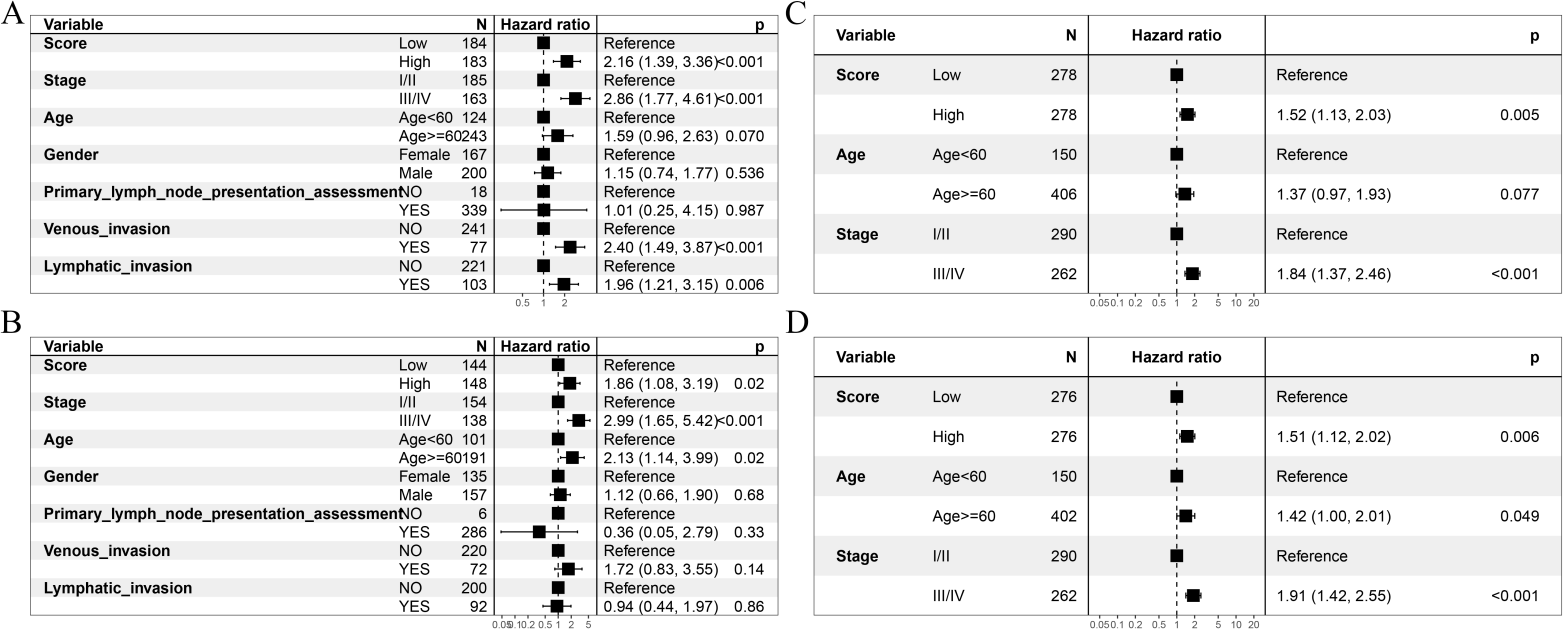
Univariate and multivariate Cox regression analyses evaluating independent prognostic factors for OS. **(A, B)** Independent prognostic value of the model’s score. **(A)** Univariate Cox analysis; **(B)** multivariate Cox analysis. **(C, D)** Independent prognostic value of the score of the validation set GSE39582. **(C)** univariate Cox analysis; **(D)** multivariate Cox analysis.

Following the successful construction and validation of the prognostic model, we performed univariate and multivariate Cox regression analyses to evaluate whether the score was an independent prognostic factor of OS. Multivariate Cox analysis confirmed that the score remained an independent OS predictor after adjusting for other confounding factors (**Figures 10A, B**) on the TCGA–COADREAD sample. The GSE39582 dataset also confirmed the score as an independent OS prognostic factor in both multivariate and multivariate Cox analyses (**Figures 10C, D**).

### 3.6 Verification of signature gene-expression results

**Figure 11:**
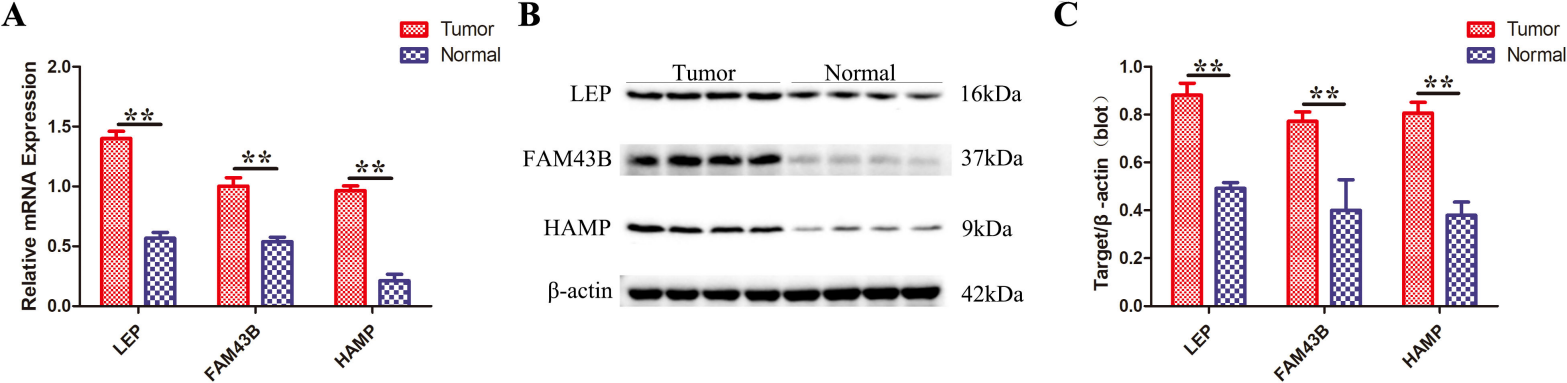
Validation of signature gene expression at mRNA and protein levels in CRC tissues compared to those in normal tissues. **(A)** RT-PCR results of the mRNA expression levels of the three signature genes. **(B)** Western blot analysis of the protein levels of the three signature genes. **(C)** Quantification of protein expression levels for these three signature genes. Data are presented as mean ± SD (*n* = 3). **P <.01.

We validated the three signature genes at both the mRNA and protein levels. The results showed that the mRNA and protein levels of LEP, HAMP, and FAM43B in CRC were higher than those in the control group, consistent with the Gene Chip data. All samples from the same experimental set and those from gels/blots were processed simultaneously (**Figure 11**).

### 3.7 Risk scoring of different clinical features

**Figure 12:**
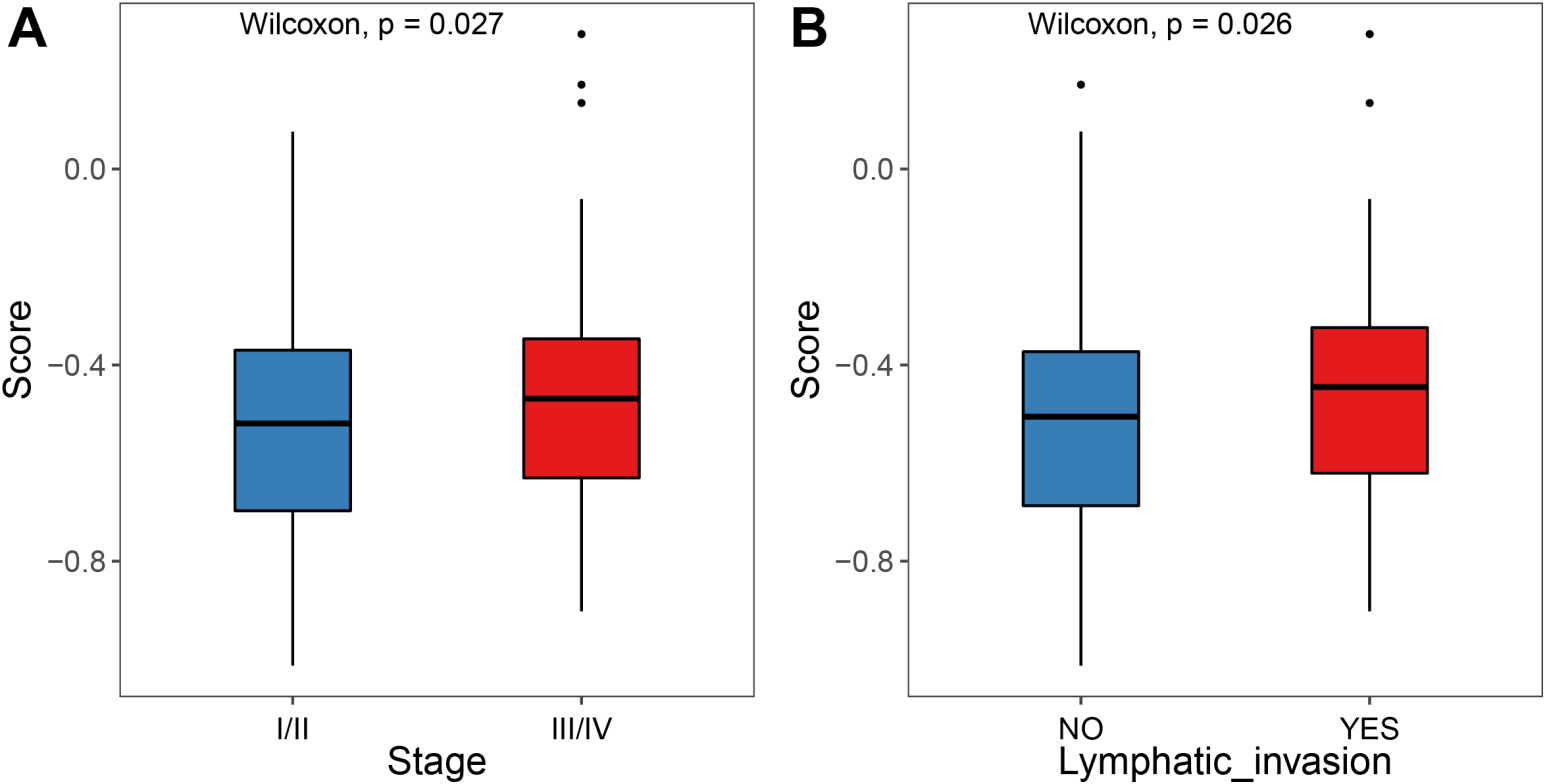
Differences in the scores of different clinical subgroups. **(A)** Comparison between risk score and stage grouping. **(B)** Comparison between risk score and lymphatic invasion status.

An analysis of the risk score for different clinical features of patients with TCGA–COADREAD. The results demonstrated significant differences in score distribution when stratified by cancer stages and lymph invasion status (**Figure 12**).

### 3.8 Molecular mechanism analysis of prognosis differences in different score groups

**Figure 13:**
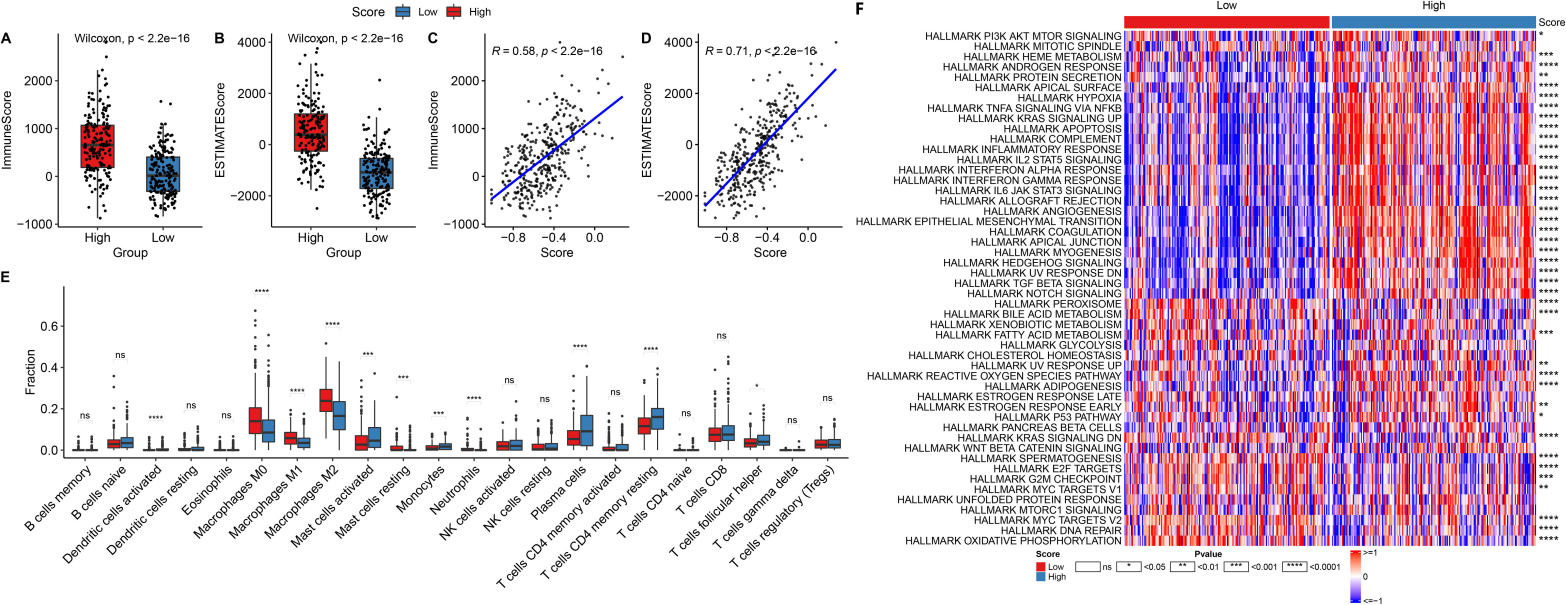
Immune cell infiltration and pathway enrichment analysis in high- and low-score groups. **(A, B)** Box plots comparing the **(A)** immune scores and **(B)** ESTIMATE scores between high- and low-score groups. **(C)** correlation between immune score and model score. **(D)** correlation between ESTIMATE score and model score. **(E)** Box plots showing the proportion of immune cell infiltration for different cell types between high-score (red) and low-score (blue) groups. **(F)** Heatmap of HALLMARK pathway enrichment scores between different model groups. *P<.05, **P<.01, ***P<.001, ****P<.0001. NS, not significant.

After the validation of the prognostic model, we re-classified the patients into two groups based on the median prognostic model score. We also used the ESTIMATE algorithm to assess the immune score and matrix score differences between the two patient groups. As shown in **Figures 13A, B**, patients with high immune scores were significantly greater than those with low immune scores. The correlation between the immune score and the prognostic model score, the ESTIMATE score, and the prognostic score is shown in **Figures 13C, D**.

The CIBERSORT algorithm was employed again to calculate the infiltration immune cell infiltration proportion for each patient and to compare differences in the tumor microenvironment (TME) between high- and low-prognostic model score groups. The infiltration degree of immune cells, particularly macrophages M0, M1, and M2, was higher in the high-prognostic model score group compared to that in the low-prognostic model score group (**Figure 13E**).

Furthermore, we compared the immune scores in both high- and low-prognostic model score groups in different HALLMARK pathways. We found that the low-prognostic model score group was positively correlated with many cancer super-pathways, including MYC targets V2, PI3K AKT MTOR signaling, and DNA repair. A few pathways were negatively correlated with the low-prognostic score group, such as TGF β signaling, NOTCH signaling, and IL6-JAK-STAT3 signaling.

In contrast, the high-prognostic model score group was positively correlated with immune response super-pathways, such as complement, IL2 STAT5 signaling, and angiogenesis. A few pathways are negatively correlated with the high-score group, such as oxidative phosphorylation, DNA repair, and MYC targets V2 (**Figure 13F**).

### 3.9 Potential treatment strategies of the model

**Figure 14:**
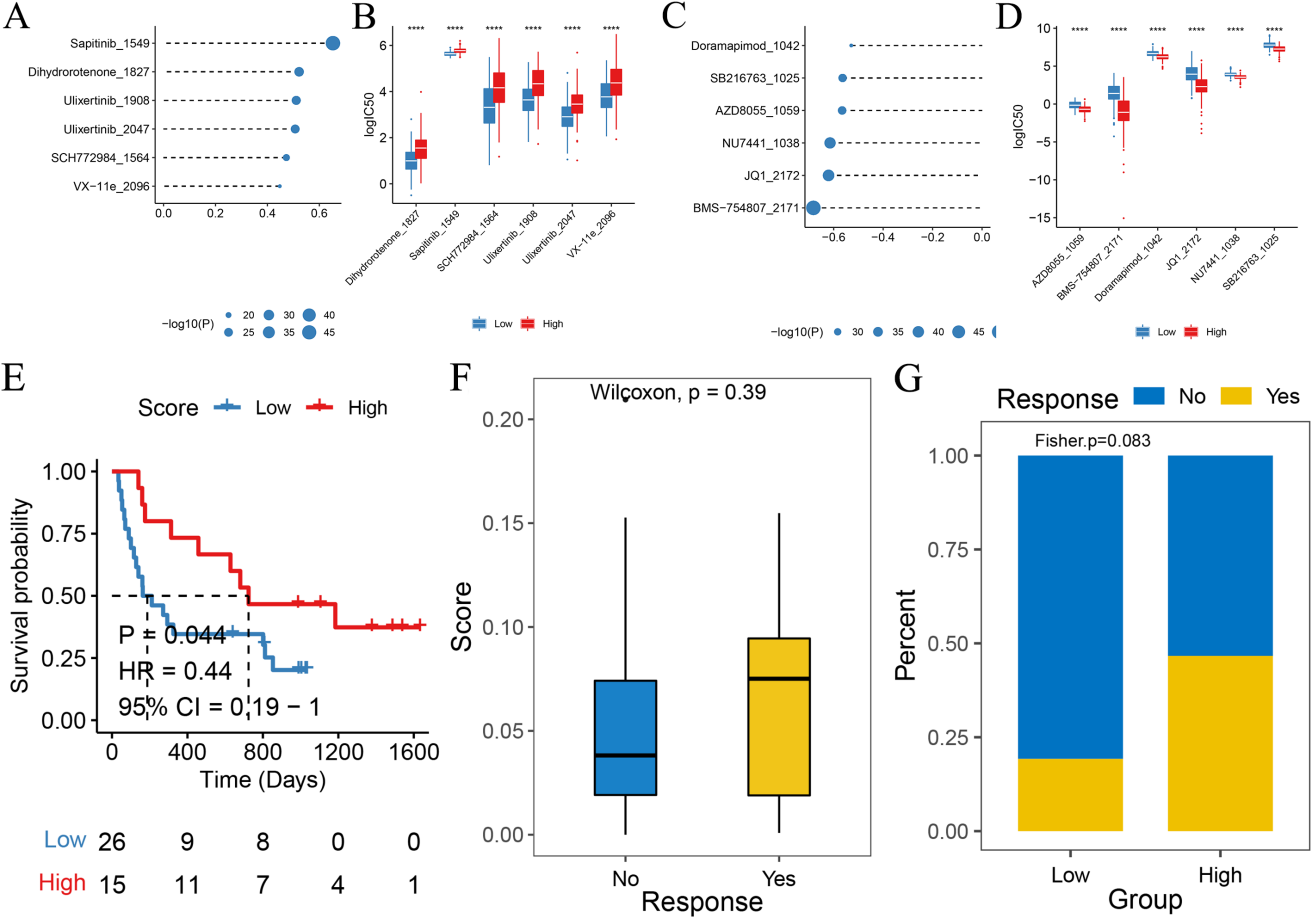
Drug sensitivity and immunology response analysis based on the model scores. **(A)** Bubble plot showing the top six drugs positively correlated with high-score groups, indicating higher resistance. **(B)** Box plots comparing log(IC50) between high-score (red) and low-score (blue) groups for the top six drugs correlated with the score. **(C)** Bubble plot showing the top six drugs negatively correlated with high-score groups, indicating higher sensitivity. **(D)** Box plot comparing log(IC50) between high-score (red) and low-score (blue) groups for the top six drugs correlated with the score. **(E)** Kaplan–Meier survival curve for immunotherapy patients in the IMvigor210 cohort, with high-score (red) and low-score (blue) groups. **(F, G)** Box plot comparing model scores between patients with complete/partial response (CR/PR) and stable/progressive disease in the IMvigor210 cohort.

We calculated the sensitivity scores of the drugs using the OncePredict package. The results revealed that Sapitinbi_1549, Dihydrorotenone_1827, Ulixertinib_1908, Ulixertinib_2047, SCH772984_ 1564, and VX-11e_2096 were the top six drugs with positive correlations to the scores, while Doramapimod_1042, SB216763_1025, AZD8055_1059, NU7441_1038, JQ_2172, and BMS-754807_2171 were the top six drugs with negative correlations (**Figures 14A–D**).

To further validate the clinical relevance of the scoring model, we evaluated its predictive efficacy in an independent immunotherapy cohort (phs000452.v2.p1). We used the same algorithm as the training set to calculate the risk score for each patient. The patients were then placed in high-score and low-score groups based on the surv-cutpoint function. A comparison of the overall survival (OS) of the two groups of patients through Kaplan–Meier survival analysis showed that patients in the low-score group had a significantly better prognosis than those in the high-score group (log rank *P* < 0.05), indicating that in our scoring model, high-score patients had improved prognosis after receiving immunotherapy. Patients insensitive to immunotherapy generally had higher scores, as shown in **Figures 14E–G**.

## 4 Discussion

By utilizing the CRC data from the TCGA database, this study identified the CRC subtypes of anoikis-related genes and developed a prognostic signature. The expression levels of CEACAM6, CHEK2, E2F1, IKBKG, NOTCH1, PTK2, PTRH2, SRC, STK11, and TSC2 in tumor tissues were significantly higher than those in adjacent tissues. Several anoikis-related genes have been implicated in tumor progression across malignancies, including CRC (30). CEACAM6, a key member of the immunoglobulin superfamily and a nonspecific cross-antigen, may play a synergistic role with various proteins such as ITGB1 and CYR62 in promoting tumor growth, proliferation, migration, and angiogenesis, contributing to CRC occurrence and development (31–33). Studies have shown that E2F1 can act both as a promoter of cell proliferation and an inhibitor of apoptosis, playing a dual role in tumor progression. High expression of E2F1 is correlated with the reduction of the tumor volume, independent of tumor location and lymph node metastasis (34). Notch 1 is overexpressed in CRC and identified as a potential carcinogen of CRC contributing to the occurrence and development of the disease (35, 36). KEGG enrichment analysis of these genes showed that these differential genes were significantly enriched in ECM–receiver interactions, neuroactive light–receiver interactions, and other similar pathways. Furthermore, we stratified CRC patients based on the anoikis-related gene expression of nest-loss apoptosis and constructed the relevant subtype identification.

We successfully constructed a prognostic model between the subtypes and the laser Cox regression analysis and verified the validity of our model in independent datasets. Patients were divided into high- and low-score groups based on the median score. The prognosis of patients in the high-score group was significantly poorer than those in the low-score group. These results indicate that the anoikis-related gene plays an important role in the pathogenesis of CRC and has a significant impact on the prognosis of CRC patients.

Tumor-infiltrating immune cells are important components of tumor tissues and play a major role in tumor initiation and progression. These immune cells reveal the heterogeneity types and diversity of the tumor and provide prognostic information for cancer patients (37). Among these, macrophages, originating from the bone marrow stem cells and differentiating under the effect of various stimulators, participate in both the innate immunity and specific immunity of the body and play an important role in the elimination of pathogenic microorganisms and tissue repair in the body (38). The CRC tumor-associated macrophages (TAM) are abundant and contribute to immunosuppression, tumor cell proliferation, angiogenesis, tumor progression, metastasis, and resistance to therapy. Both antitumor M1 macrophages and tumor-promoting M2 macrophages coexist in the TME and their interactions directly influence CRC progression and clinical treatment strategies (39). M2 macrophages produce growth-promoting factors that stimulate tumor growth in a way that promotes healing as well. However, it should be noted that the transition of M2 macrophages to M1 macrophages can decelerate tumor progression (40). One of the mechanisms of immune escape is that the tumor reprograms macrophage metabolism to prevent M1 macrophage-mediated inflammatory reaction from increasing the killing of tumor cells. Although the macrophages in the CRC TME are not completely M1 and M2 type, M2 macrophages are usually more prevalent and induce immunosuppression to promote tumor growth (41). In this study, we obtained bulk RNA-seq and microarray data of CRC and employed CIBERSORT algorithm to compare the differences in immune cells between high- and low-score groups. The results showed that the proportion of M0, M1, and M2 macrophages in the high-score group was significantly higher than that in the low-score group. In contrast, the proportions of CD4 memory-activated T cells, activated mast cells, and plasma cells were lower in the high-score group than they were in the low-score group, suggesting that macrophage infiltration plays a critical role in CRC progression. These observations indicate that the anoikis-related genes may affect the microenvironment of CRC by regulating macrophages, leading to the occurrence of CRC.

Despite the rapid development of laparoscopic CRC radical surgery, such as total mesorectal resection and total mesocolon resection, the median survival rate of CRC patients is still low because many CRC patients are diagnosed at advanced stages, resulting in poor treatment outcomes. In recent years, however, the development of oncology, immunology, molecular biology, and other related disciplines has led to the emergence of immunotherapy as an important treatment method for CRC. Immunotherapy can eliminate tumors by inhibiting negative immune regulators, activating the immune system, and enhancing the recognition and killing of immune cells to tumors. In the immunotherapy cohort, the prognosis of patients with high scores significantly improved, suggesting that the model can provide a reference for possible subsequent immunotherapy. We predicted drug sensitivity using the GDSC2 database and found that Sapitinib_1549, Dihydrorotenone_1827, Doramapimod_1042, SB216763_1025, and other drugs can be used for high-sensitivity CRC treatment, offering potential theoretical options and potential therapeutic targets for the clinical drug treatment of CRC in the future.

Our study successfully constructed a prognostic model by using differential genes between the subtypes. The genes were placed in independent datasets using the Lasso Cox analysis method and the patients were divided into high- and low-score groups based on their median score. The prognosis of patients with a high score was significantly worse than those in the low-score group. In addition, we observed significant differences in the immune microenvironment infiltration between the two groups of patients and found that the high-score group had a higher infiltration of macrophages M0, M1, and M2 than that in the low-score group. In contrast, in the immunotherapy cohort, the prognosis of patients with high scores, as judged by the model, was significantly better than that of the patients with low scores, suggesting the possibility of developing a possible immunotherapy. Additionally, drug sensitivity can be predicted using the GDSC2 database. This study also identified anoikis-related gene subtypes and developed a prognostic signature in CRC, laying a foundation for further investigations of the molecular mechanism, clinical diagnosis, and treatment of CRC.

However, our study still has some limitations. We used algorithm analyses to predict the molecular subtypes and prognostic models of anoikis-related genes in CRC in the public database. However, we did not verify it in our patient cohort. Future work will aim to collect more specimens for multi-omics analysis to validate our findings. In addition, the relevant molecular mechanisms were not experimentally verified in this study. Therefore, future research experiments should investigate and validate the regulatory mechanism underlying cell-cell communication through ligand–receptor interactions that contribute to CRC pathogenesis using functional experiments and appropriate animal models.

## 5 Conclusions

This study has significance for the exploration of the molecular subtypes and prognostic models of anoikis-related genes in CRC. Our findings enhance the understanding of the molecular mechanisms underlying CRC initiation and progression. Moreover, this study offers a unique approach to the discovery of predictive biomarkers and the selection of targeted therapy for CRC treatment. We believe that the prognostic model discussed in our study demonstrates strong stability and generalizability.

## Data Availability

The datasets presented in this study can be found in online repositories. The names of the repository/repositories and accession number(s) can be found in the article/**Supplementary Material**.

## References

[1] Malki A ElRuz RA Gupta I Allouch A Vranic S Al Moustafa AE. Molecular mechanisms of colon cancer progression and metastasis: recent insights and advancements. Int J Mol Sci. (2020) 22:130. doi: 10.3390/ijms22010130 33374459 PMC7794761

[2] Panza A Castellana S Biscaglia G Piepoli A Parca L Gentile A. Transcriptome and gene fusion analysis of synchronous lesions reveals lncMRPS31P5 as a novel transcript involved in colorectal cancer. Int J Mol Sci. (2020) 21:7120. doi: 10.3390/ijms21197120 32992457 PMC7582694

[3] Codrich M Dalla E Mio C Antoniali G Malfatti MC Marzinotto S. Integrated multi-omics analyses on patient-derived CRC organoids highlight altered molecular pathways in colorectal cancer progression involving PTEN. J Exp Clin Cancer Res. (2021) 40:198. doi: 10.1186/s13046-021-01986-8 34154611 PMC8215814

[4] Pecci F Cantini L Bittoni A Lenci E Lupi A Crocetti S. Beyond microsatellite instability: evolving strategies integrating immunotherapy for microsatellite stable colorectal cancer. Curr Treat Options Oncol. (2021) 22:69. doi: 10.1007/s11864-021-00870-z 34110510 PMC8192371

[5] Shaukat A Kahi CJ Burke CA Rabeneck L Sauer BG Rex DK. ACG clinical guidelines: colorectal cancer screening 2021. Am J Gastroenterol. (2021) 116:458–79. doi: 10.14309/ajg.0000000000001122 33657038

[6] Thanikachalam K Khan G. Colorectal cancer and nutrition. Nutrients. (2019) 11:164. doi: 10.3390/nu11010164 30646512 PMC6357054

[7] Poturnajova M Furielova T Balintova S Schmidtova S Kucerova L Matuskova M. Molecular features and gene expression signature of metastatic colorectal cancer (Review). Oncol Rep. (2021) 45:10. doi: 10.3892/or.2021.7961 33649827 PMC7876998

[8] Biller LH Schrag D. Diagnosis and treatment of metastatic colorectal cancer: A review. JAMA. (2021) 325:669–85. doi: 10.1001/jama.2021.0106 33591350

[9] Andrei P Battuello P Grasso G Rovera E Tesio N Bardelli A. Integrated approaches for precision oncology in colorectal cancer: The more you know, the better. Semin Cancer Biol. (2022) 84:199–213. doi: 10.1016/j.semcancer.2021.04.007 33848627

[10] Frisch SM Francis H. Disruption of epithelial cell-matrix interactions induces apoptosis. J Cell Biol. (1994) 124:619–26. doi: 10.1083/jcb.124.4.619 PMC2119917 8106557

[11] Grossmann J. Molecular mechanisms of “detachment-induced apoptosis–Anoikis. Apoptosis. (2002) 7:247–60. doi: 10.1023/A:1015312119693 11997669

[12] Deng D Wu Y Wu K Zeng N Li W. Dihydroberberine alleviates Th17/Treg imbalance in premature ovarian insufficiency mice via inhibiting Rheb/mTOR signaling. Mol Med. (2024) 30:194. doi: 10.1186/s10020-024-00971-z 39472803 PMC11523677

[13] Liu J Lichtenberg T Hoadley KA Poisson LM Lazar AJ Cherniack AD. An integrated TCGA pan-cancer clinical data resource to drive high-quality survival outcome analytics. Cell. (2018) 173:400–16.e11. doi: 10.1016/j.cell.2018.02.052 29625055 PMC6066282

[14] Huber W Carey VJ Gentleman R Anders S Carlson M Carvalho BS. Orchestrating high-throughput genomic analysis with Bioconductor. Nat Methods. (2015) 12:115–21. doi: 10.1038/nmeth.3252 PMC4509590 25633503

[15] Bindea G Mlecnik B Tosolini M Kirilovsky A Waldner M Obenauf AC. Spatiotemporal dynamics of intratumoral immune cells reveal the immune landscape in human cancer. Immunity. (2013) 39:782–95. doi: 10.1016/j.immuni.2013.10.003 24138885

[16] Yu G Wang LG Han Y He QY. clusterProfiler: an R package for comparing biological themes among gene clusters. Omics. (2012) 16:284–7. doi: 10.1089/omi.2011.0118 PMC3339379 22455463

[17] Chengmao X Li L Yan L Jie Y Xiaoju W Xiaohui C. ABCA1 affects placental function via trophoblast and macrophage. Life Sci. (2017) 191:150–6. doi: 10.1016/j.lfs.2017.10.031 29066252

[18] Subramanian A Tamayo P Mootha VK Mukherjee S Ebert BL Gillette MA. Gene set enrichment analysis: a knowledge-based approach for interpreting genome-wide expression profiles. Proc Natl Acad Sci U S A. (2005) 102:15545–50. doi: 10.1073/pnas.0506580102 PMC1239896 16199517

[19] Mootha VK Lindgren CM Eriksson KF Subramanian A Sihag S Lehar J. PGC-1alpha-responsive genes involved in oxidative phosphorylation are coordinately downregulated in human diabetes. Nat Publishing Group. (2003) 34(3):267–73. doi: 10.1038/ng1180 12808457

[20] Ferreira MR Santos GA Biagi CA Silva Junior WA Zambuzzi WF. GSVA score reveals molecular signatures from transcriptomes for biomaterials comparison. J BioMed Mater Res A. (2021) 109:1004–14. doi: 10.1002/jbm.a.37090 32820608

[21] Cibulskis K Lawrence MS Carter SL Sivachenko A Jaffe D Sougnez C. Sensitive detection of somatic point mutations in impure and heterogeneous cancer samples. Nat Biotechnol. (2013) 31:213–9. doi: 10.1038/nbt.2514 PMC3833702 23396013

[22] Kawada JI Takeuchi S Imai H Okumura T Horiba K Suzuki T. Immune cell infiltration landscapes in pediatric acute myocarditis analyzed by CIBERSORT. J Cardiol. (2021) 77:174–8. doi: 10.1016/j.jjcc.2020.08.004 32891480

[23] Iorio F Knijnenburg TA Vis DJ Bignell GR Menden MP Schubert M. A landscape of pharmacogenomic interactions in cancer. Cell. (2016) 166:740–54. doi: 10.1016/j.cell.2016.06.017 PMC4967469 27397505

[24] Maeser D Gruener RF Huang RS. oncoPredict: an R package for predicting *in vivo* or cancer patient drug response and biomarkers from cell line screening data. Brief Bioinform. (2021) 22:bbab260. doi: 10.1093/bib/bbab260 34260682 PMC8574972

[25] Mariathasan S Turley SJ Nickles D Castiglioni A Yuen K Wang Y. TGFβ attenuates tumour response to PD-L1 blockade by contributing to exclusion of T cells. Nature. (2018) 554:544–8. doi: 10.1038/nature25501 PMC6028240 29443960

[26] Zeng D Ye Z Shen R Yu G Wu J Xiong Y. IOBR: multi-omics immuno-oncology biological research to decode tumor microenvironment and signatures. Front Immunol. (2021) 12:687975. doi: 10.3389/fimmu.2021.687975 34276676 PMC8283787

[27] Gu Z. Complex heatmap visualization. iMeta. (2022) 1(3):e43. doi: 10.1002/imt2.43 38868715 PMC10989952

[28] Couch S Bray A Ismay C Chasnovski E Baumer B Etinkaya-Rundel M. infer: An R package for tidyverse-friendly statistical inference. J Open Source Software. (2021) 6:3661. doi: 10.21105/joss.03661

[29] Gu Z Huebschmann D. Make interactive complex heatmaps in R. Bioinformatics. (2021) 38(5):1460–2. doi: 10.1101/2021.03.08.434289 PMC8826183 34864868

[30] Sica VP Krivos KL Kiehl DE Pulliam CJ Henry ID Baker TR. The role of mass spectrometry and related techniques in the analysis of extractable and leachable chemicals. Mass Spectrom Rev. (2020) 39:212–26. doi: 10.1002/mas.21591 30921495

[31] Gu A Tsark W Holmes KV Shively JE. Role of Ceacam1 in VEGF induced vasculogenesis of murine embryonic stem cell-derived embryoid bodies in 3D culture. Exp Cell Res. (2009) 315:1668–82. doi: 10.1016/j.yexcr.2009.02.026 PMC2745895 19285068

[32] Tilki D Singer BB Shariat SF Behrend A Fernando M Irmak S. CEACAM1: a novel urinary marker for bladder cancer detection. Eur Urol. (2010) 57:648–54. doi: 10.1016/j.eururo.2009.05.040 19487071

[33] Belvedere O Puglisi F Di Loreto C Cataldi P Guglielmi A Aschele C. Lack of correlation between immunohistochemical expression of E2F-1, thymidylate synthase expression and clinical response to 5-fluorouracil in advanced colorectal cancer. Ann Oncol. (2004) 15:55–8. doi: 10.1093/annonc/mdh018 14679120

[34] Chu D Li Y Wang W Zhao Q Li J Lu Y. High level of Notch1 protein is associated with poor overall survival in colorectal cancer. Ann Surg Oncol. (2010) 17:1337–42. doi: 10.1245/s10434-009-0893-7 20058190

[35] Qiu S Nikolaou S Zhu J Jeffery P Goldin R Kinross J. Characterisation of the expression of neurotensin and its receptors in human colorectal cancer and its clinical implications. Biomolecules. (2020) 10:1145. doi: 10.3390/biom10081145 32764278 PMC7464404

[36] Angell H Galon J. From the immune contexture to the Immunoscore: the role of prognostic and predictive immune markers in cancer. Curr Opin Immunol. (2013) 25:261–7. doi: 10.1016/j.coi.2013.03.004 23579076

[37] Artyomov MN Sergushichev A Schilling JD. Integrating immunometabolism and macrophage diversity. Semin Immunol. (2016) 28:417–24. doi: 10.1016/j.smim.2016.10.004 PMC5333784 27771140

[38] Boutilier AJ Elsawa SF. Macrophage polarization states in the tumor microenvironment. Int J Mol Sci. (2021) 22:6995. doi: 10.3390/ijms22136995 34209703 PMC8268869

[39] Van der Jeught K Xu HC Li YJ Lu XB Ji G. Drug resistance and new therapies in colorectal cancer. World J Gastroenterol. (2018) 24:3834–48. doi: 10.3748/wjg.v24.i34.3834 PMC6141340 30228778

[40] Wu K Lin K Li X Yuan X Xu P Ni P. Redefining tumor-associated macrophage subpopulations and functions in the tumor microenvironment. Front Immunol. (2020) 11:1731. doi: 10.3389/fimmu.2020.01731 32849616 PMC7417513

[41] Mills CD Lenz LL Harris RA. A breakthrough: macrophage-directed cancer immunotherapy. Cancer Res. (2016) 76:513–6. doi: 10.1158/0008-5472.CAN-15-1737 PMC4738030 26772756

