# Supplementary figures and images for: Analysis of the molecular subtypes and prognostic models of anoikis-related genes in colorectal cancer
Source: Front Oncol. 2025 Jun 30;15:1579843. doi: 10.3389/fonc.2025.1579843 (PMC12256226; doi:10.3389/fonc.2025.1579843)

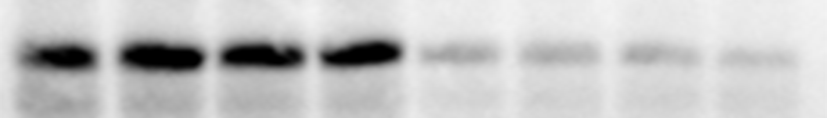

Supplement: Supplementary file 1 [file DataSheet1.zip › The original image of Figure 11/FAM43B.tif]

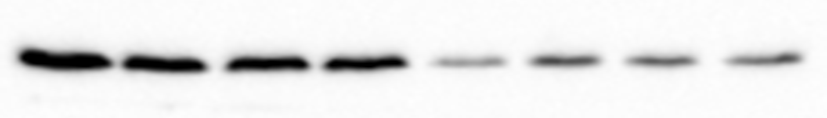

Supplement: Supplementary file 1 [file DataSheet1.zip › The original image of Figure 11/HAMP.tif]

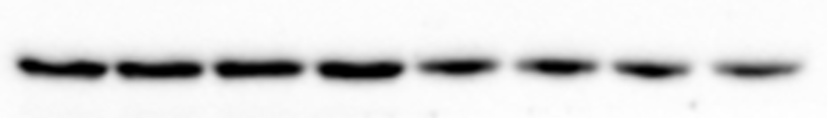

Supplement: Supplementary file 1 [file DataSheet1.zip › The original image of Figure 11/LEP.tif]

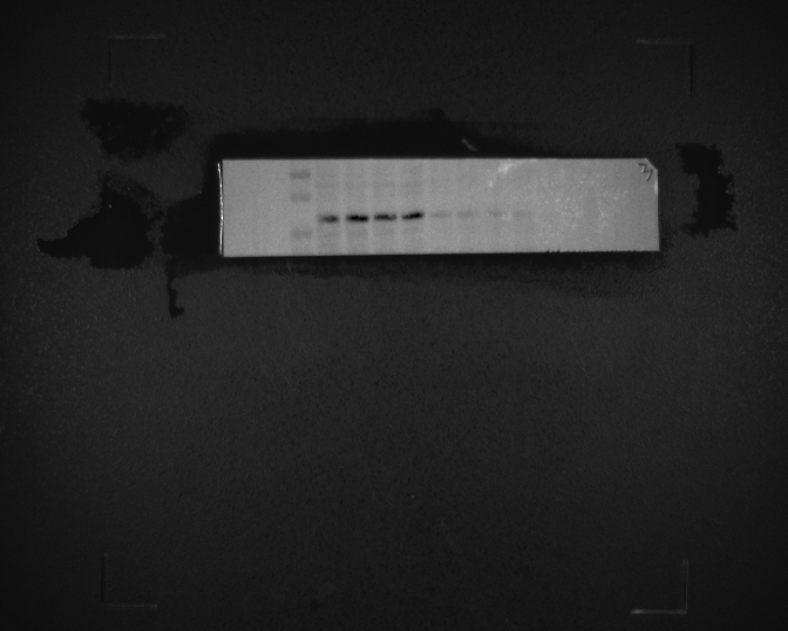

Supplement: Supplementary file 1 [file DataSheet1.zip › The original image of Figure 11/microscopy images-FAM43B.tif]

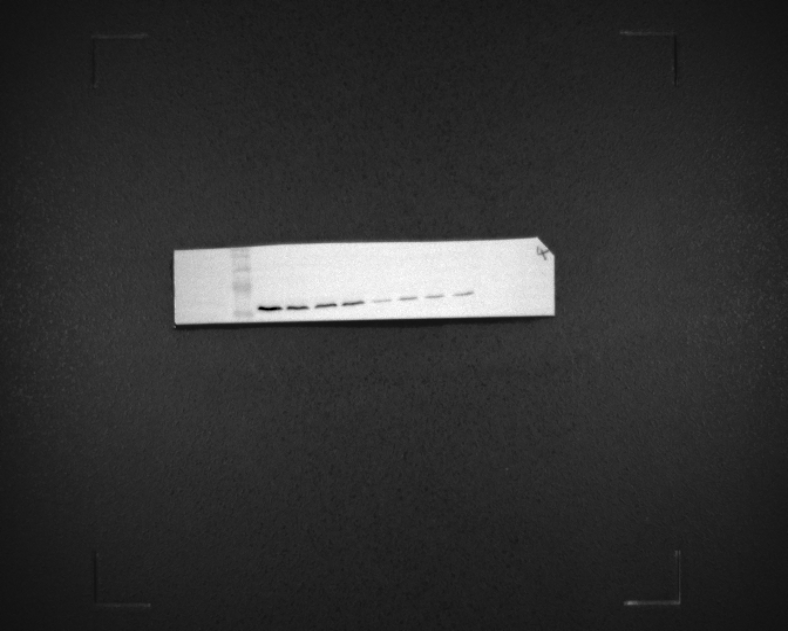

Supplement: Supplementary file 1 [file DataSheet1.zip › The original image of Figure 11/microscopy images-HAMP.tif]

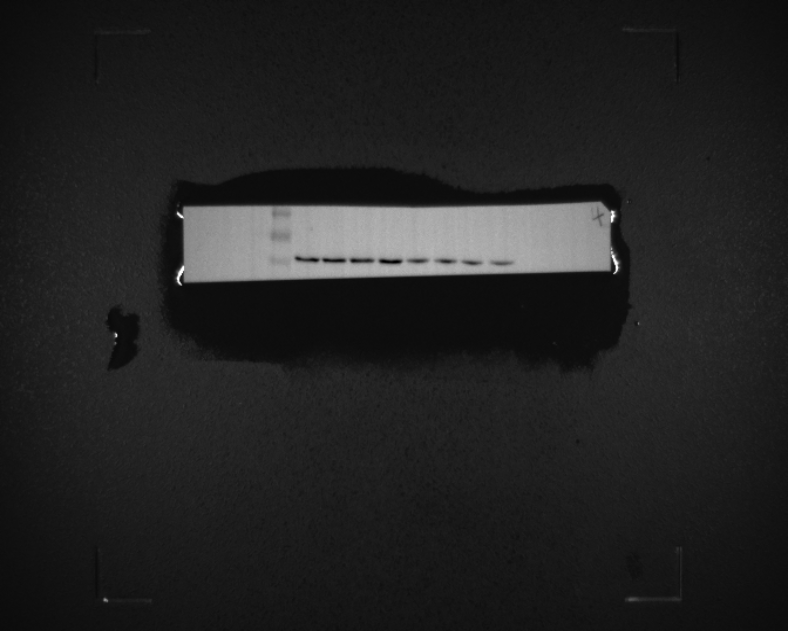

Supplement: Supplementary file 1 [file DataSheet1.zip › The original image of Figure 11/microscopy images-LEP.tif]

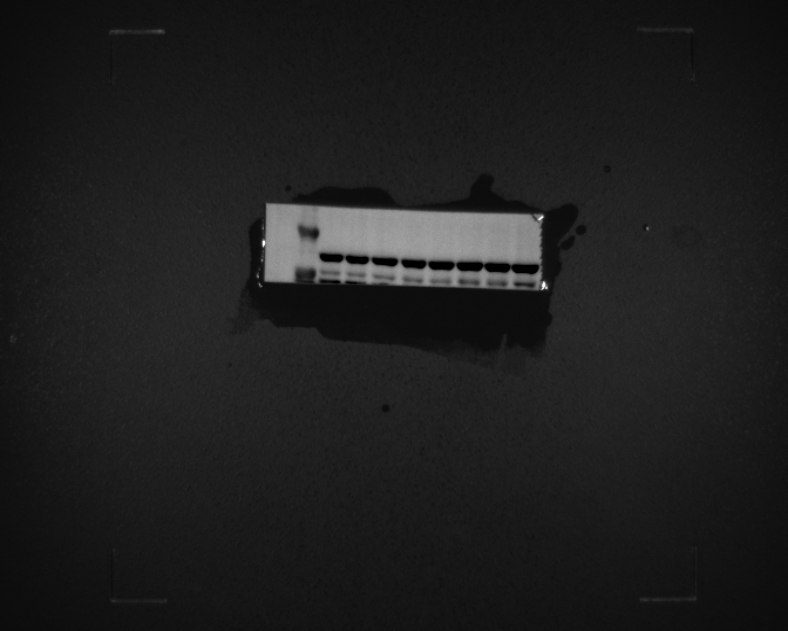

Supplement: Supplementary file 1 [file DataSheet1.zip › The original image of Figure 11/microscopy images-β-actin.tif]

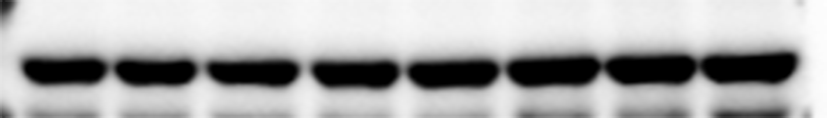

Supplement: Supplementary file 1 [file DataSheet1.zip › The original image of Figure 11/β-actin.tif]

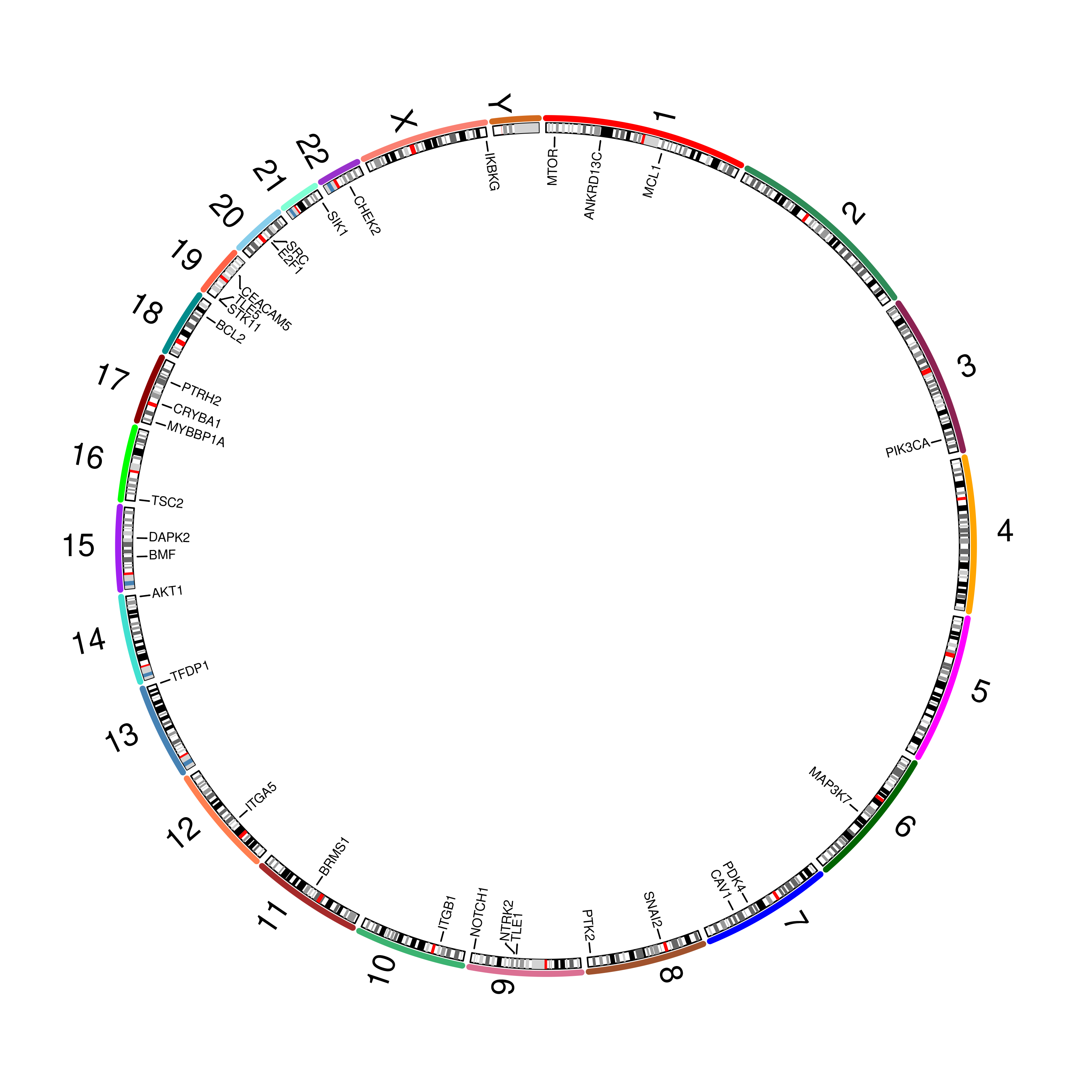

Supplement: Supplementary file 2 [file Image1.tif]

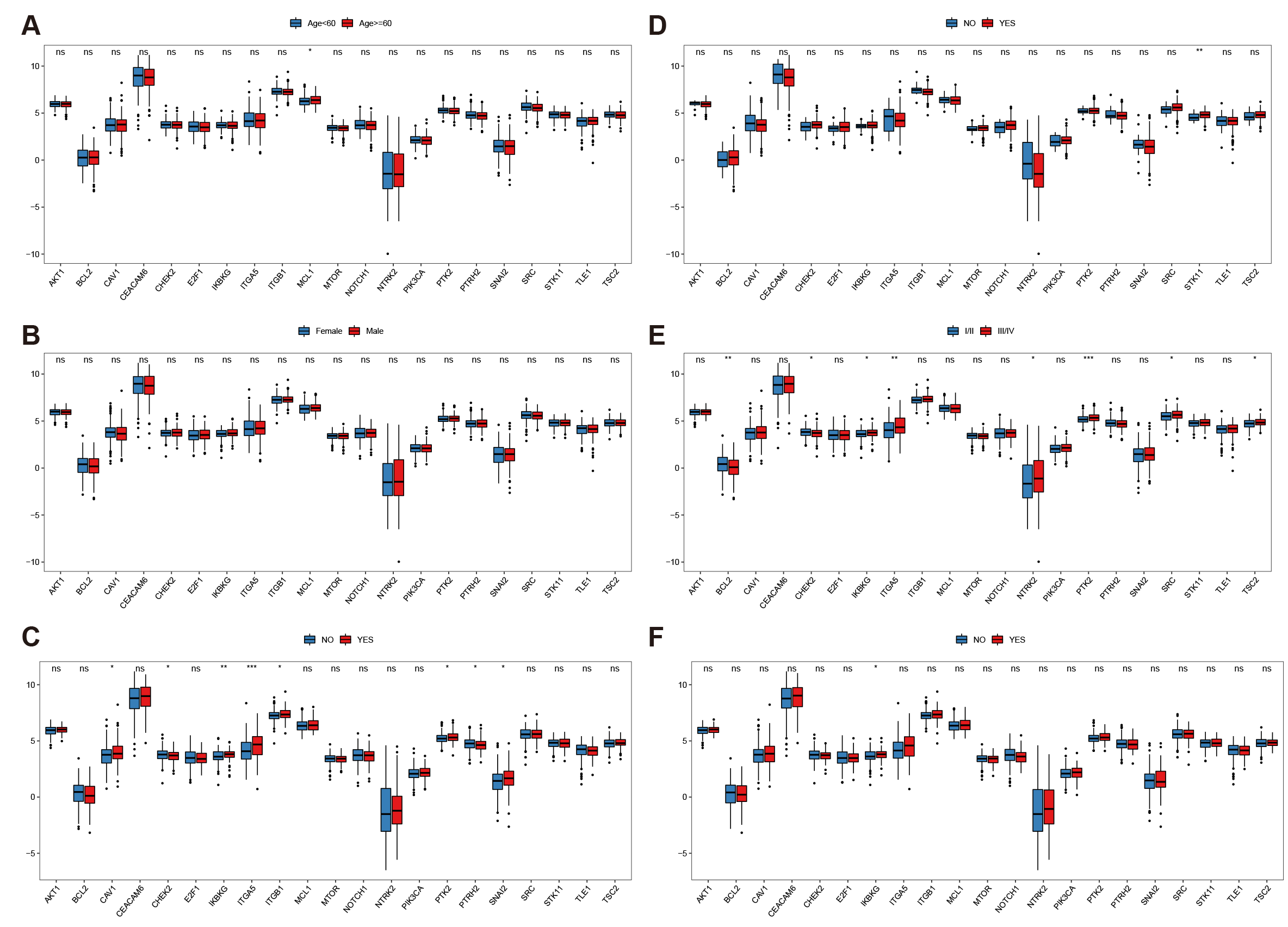

Supplement: Supplementary file 3 [file Image2.tif]

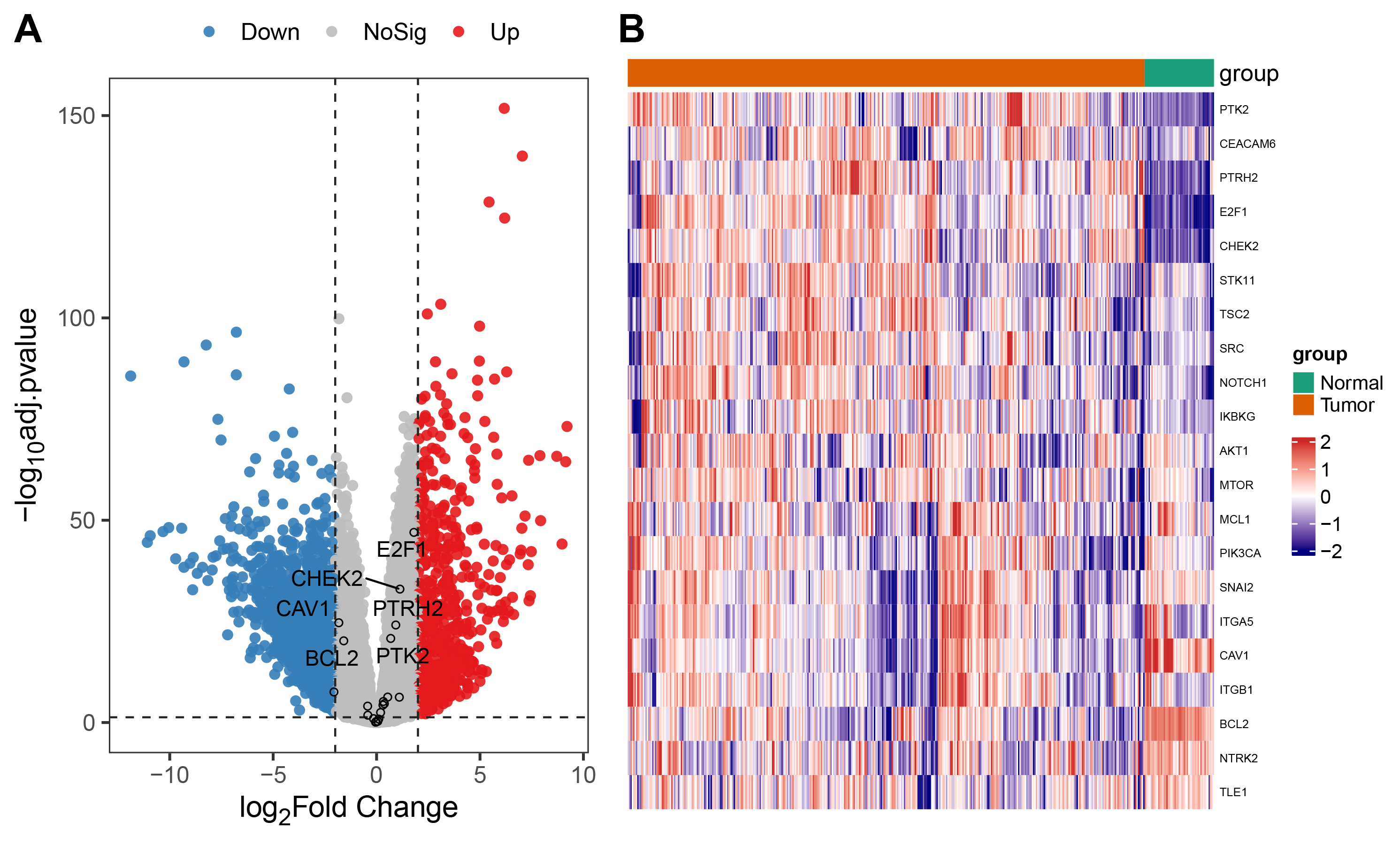

Supplement: Supplementary file 4 [file Image3.tif]

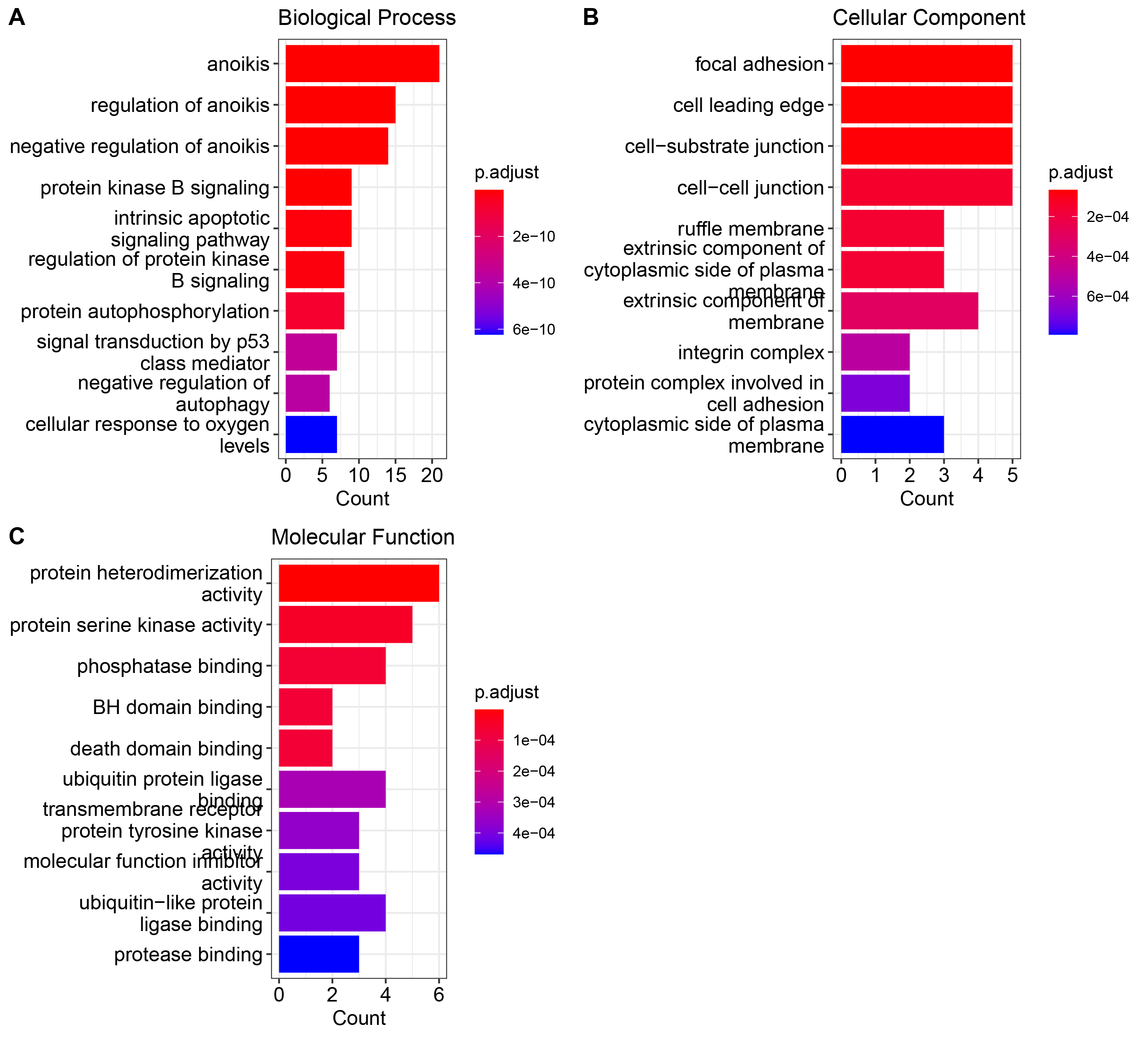

Supplement: Supplementary file 5 [file Image4.tif]
